# Genome sequence of *Gossypium herbaceum* and genome updates of *Gossypium arboreum* and *Gossypium hirsutum* provide insights into cotton A-genome evolution

**DOI:** 10.1038/s41588-020-0607-4

**Published:** 2020-04-13

**Authors:** Gai Huang, Zhiguo Wu, Richard G. Percy, Mingzhou Bai, Yang Li, James E. Frelichowski, Jiang Hu, Kun Wang, John Z. Yu, Yuxian Zhu

**Affiliations:** 1 Institute for Advanced Studies, Wuhan University, Wuhan, China; 2 State Key Laboratory of Protein and Plant Gene Research, School of Life Sciences, Peking University, Beijing, China; 3 College of Life Sciences, Wuhan University, Wuhan, China; 4 Crop Germplasm Research Unit, Southern Plains Agricultural Research Center, United States Department of Agriculture-Agricultural Research Service (USDA-ARS), College Station, TX USA; 5 BGI Genomics, BGI-Shenzhen, Shenzhen, China; 6 Nextomics Biosciences Institute, Wuhan, China

**Keywords:** Sequencing, Plant sciences, Genomics

## Abstract

Upon assembling the first *Gossypium herbaceum* (A_1_) genome and substantially improving the existing *Gossypium arboreum* (A_2_) and *Gossypium hirsutum* ((AD)_1_) genomes, we showed that all existing A-genomes may have originated from a common ancestor, referred to here as A_0_, which was more phylogenetically related to A_1_ than A_2_. Further, allotetraploid formation was shown to have preceded the speciation of A_1_ and A_2_. Both A-genomes evolved independently, with no ancestor–progeny relationship. Gaussian probability density function analysis indicates that several long-terminal-repeat bursts that occurred from 5.7 million years ago to less than 0.61 million years ago contributed compellingly to A-genome size expansion, speciation and evolution. Abundant species-specific structural variations in genic regions changed the expression of many important genes, which may have led to fiber cell improvement in (AD)_1_. Our findings resolve existing controversial concepts surrounding A-genome origins and provide valuable genomic resources for cotton genetic improvement.

## Abstract (web summary)

Assembly of the first *Gossypium herbaceum* genome and improved *Gossypium arboreum* and *Gossypium hirsutum* genomes provide insights into the phylogenetic relationships and origin history of cotton A-genomes.

## Main

Cultivated cotton is one of the most economically important crop plants in the world. The allotetraploid Upland cotton, *G. hirsutum* (*n* = 2*x* = 26, (AD)_1_), currently dominates the world’s cotton commerce^1,2^. Hybridization between the Old World A-genome progenitor and a New World D-genome ancestor, followed by chromosome doubling, formed the allopolyploid cotton ~1−2 million years ago (Ma)^3,4^. Uncertainty regarding the actual A-genome donor of the widely cultivated allotetraploid cotton *G. hirsutum* has persisted^5–13^. A_1_ (*n* = *x* = 13) and A_2_ (*n* = *x* = 13), commonly known as African and Asiatic cotton, respectively, are the only two extant diploid A-genome species in the world^14^. Stephens first proposed in *Nature*, using genetic and morphological evidence, that A_2_ was the A-genome donor of present-day allopolyploid cottons^6^. Gerstel argued via cytogenetic studies that A_1_ was more closely related to the A-genome in the allopolyploids than A_2_ (ref. ^8^). Despite recent efforts to sequence the cotton genomes, including *Gossypium* *raimondii* (D_5_)^15,16^, A_2_ (refs. ^17,18^), (AD)_1_ (refs. ^10,19–21^) and *Gossypium* *barbadense*^10,21^ ((AD)_2_, a much less cultivated tetraploid cotton), the origin history of the A-genome donor for the tetraploid (AD)_1_-genome^5,11,13^ and the extent of divergence between the A-genomes remain elusive^22,23^. Abundant studies support a *Gossypium* species resembling D_5_ as the D-genome donor^13^, but currently there is no solid evidence to suggest that the actual A-genome donor of tetraploid cottons is either A_2_ (refs. ^6,7,10,19^) or A_1_ (refs. ^8,9,11–13^) as has been suggested.

In this study, we assembled A_1_ variety *africanum* for the first time and re-assembled high-quality A_2_ cultivar Shixiya1 and (AD)_1_ genetic standard Texas Marker-1 (TM-1) genomes on the basis of PacBio long reads, paired-end sequencing and high-throughput chromosome conformation capture (Hi-C) technologies. Upon assembling and updating cotton genomes, we revealed the origin of cotton A-genomes, the occurrence of several transposable element (TE) bursts and the genetic divergence of diploid A-genomes worldwide. Also, we identified abundant structural variations (SVs) that have affected the expression of neighboring genes and help explain phenotypic differences among the cotton species.

## Results

### Sequencing and assembly of three high-quality cotton genomes

Here we sequenced the A_1_-genome var. *africanum* for the first time by generating ~225-gigabase (Gb) PacBio single-molecule real-time (SMRT) long reads (the N50 (minimum length to cover 50% of the total length) of these reads was 13 kilobases (kb)) with 138-fold genome coverage. We generated an assembly that captured 1,556 megabases (Mb) of genome sequences, consisting of 1,781 contigs with the N50 of these contigs reaching up to 1,915 kb (Table 1). The initial assemblies were then corrected by using highly accurate Illumina paired-end reads (Supplementary Table 1). Finally, 95.69% of total contigs spanning 1,489 Mb were categorized and ordered into 13 chromosome-scale scaffolds using Hi-C data (Table 1 and Supplementary Table 1).

**Table 1:** Assembly and annotation of A_1_-, A_2_- and (AD)_1_-genomes in the current and two previous studies

Category	A_1_-genome^a^	A_2_-genome		(AD)_1_-genome	
		Ref. ^18^	Current	Ref. ^21^	Current
Total PacBio reads (Gb)	225	–	310	–	205
No. of total contigs	1,781	8,223	2,432	4,791	1,235
N50 of contigs (kb)	1,915	1,100	1,832	1,892	5,020
Anchored contigs (Mb)	1,489	1,573	1,509	2,233	2,271
No. of total scaffolds	732	4,516	1,269	2,190	342
Total assembled size (Mb)	1,556	1,710	1,637	2,347^b^	2,290

^a^A_1_-genome is assembled for the first time in this work.

^b^This genome contains 65.29 Mb ambiguous ‘N’ (unknown nucleotide) bases.

**Fig. 1:**
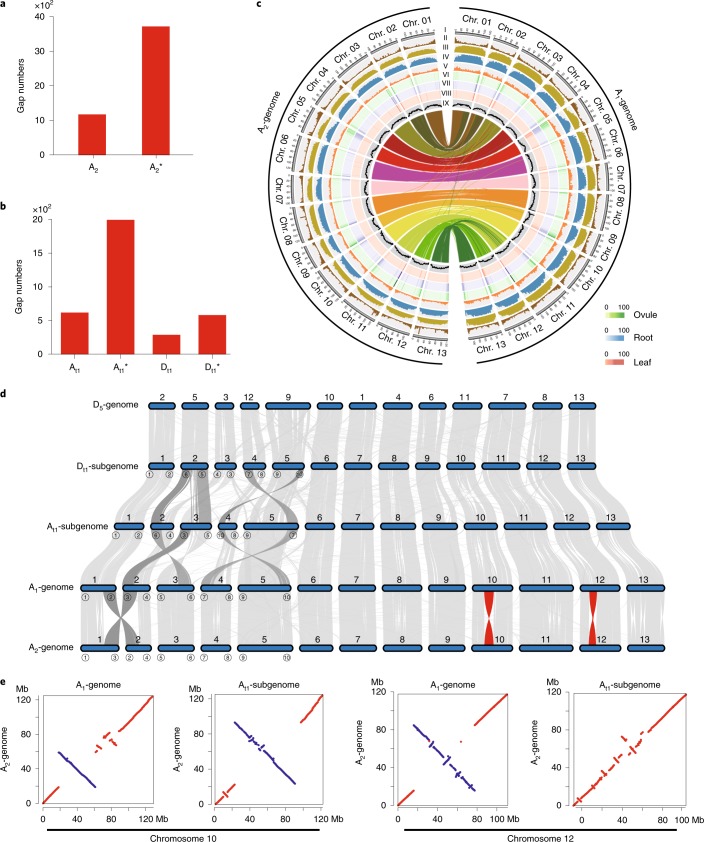
Distribution of genomic components of A_1_ and A_2_ across chromosomes and chromosomal variant events within the *Gossypium* lineage. **a**,**b**, Statistics of gap numbers in the assembly of A_2_- (**a**) and (AD)_1_- (**b**) genomes. A_2_*, previously released A_2_-genome^18^; A_t1_* and D_t1_* represent the A_t1_- and D_t1_-subgenome, respectively, of recently released (AD)_1_-genome^21^. **c**, Multi-dimensional display of genomic components of A_1_- and A_2_-genomes. The density was calculated per 1 Mb. I, the 13 chromosomes; II, gene density; III−V, coverage by TE, Gypsy and Copia, respectively; VI−VIII, transcriptional state in the ovule at 10 DPA and in root and leaf tissue, respectively. Transcript levels were estimated based on the average depth of mapped RNA reads in nonoverlapping 1-Mb windows. IX, GC content. **d**, Characterization of genomic variations in *Gossypium*. Genic synteny blocks are connected by gray lines. Reciprocal translocations and two large inversions are highlighted by dark gray and red links, respectively. **e**, Synteny maps using whole-genome alignments show that the inversion in chromosome 10 exists in either A_1_ or A_t1_, whereas the one in chromosome 12 is found only in A_1_. Genomic homologous blocks ≥ 20 kb are drawn in the plots. Chr, chromosome.

**Extended Data Fig. 1:**
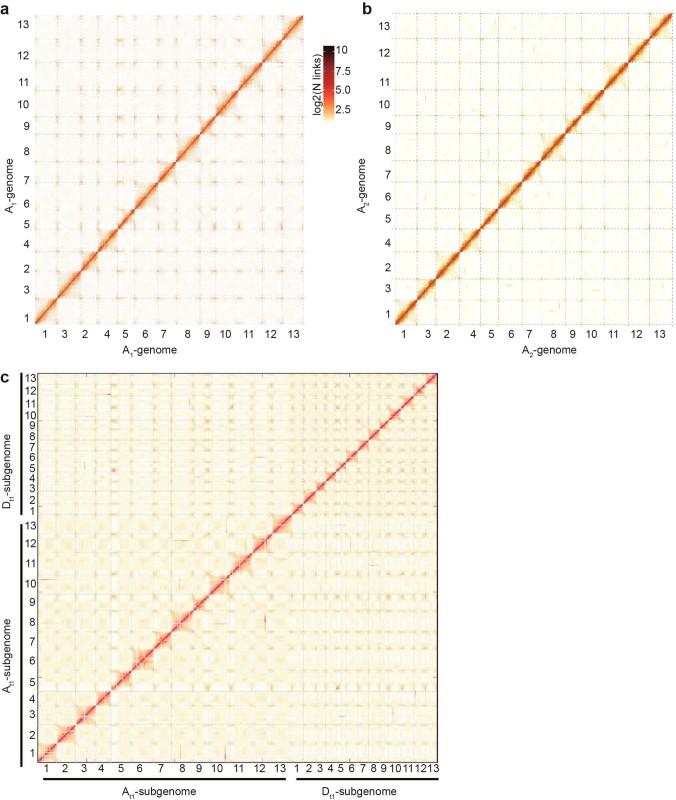
High correlation of chromosome-scale assembled A_1_, A_2_ and (AD)_1_ genomes with Hi-C data. **a**, Hi-C contact data from A_1_ mapped on the assembled A_1_-genome. **b**, Hi-C contact data from A_2_ mapped on the improved A_2_-genome. **c**, Hi-C contact data from (AD)_1_ mapped on the improved (AD)_1_-genome. The heat map represents the normalised contact matrix. The strongest and weakest contacts are shown in red and grey, respectively.

**Extended Data Fig. 2:**
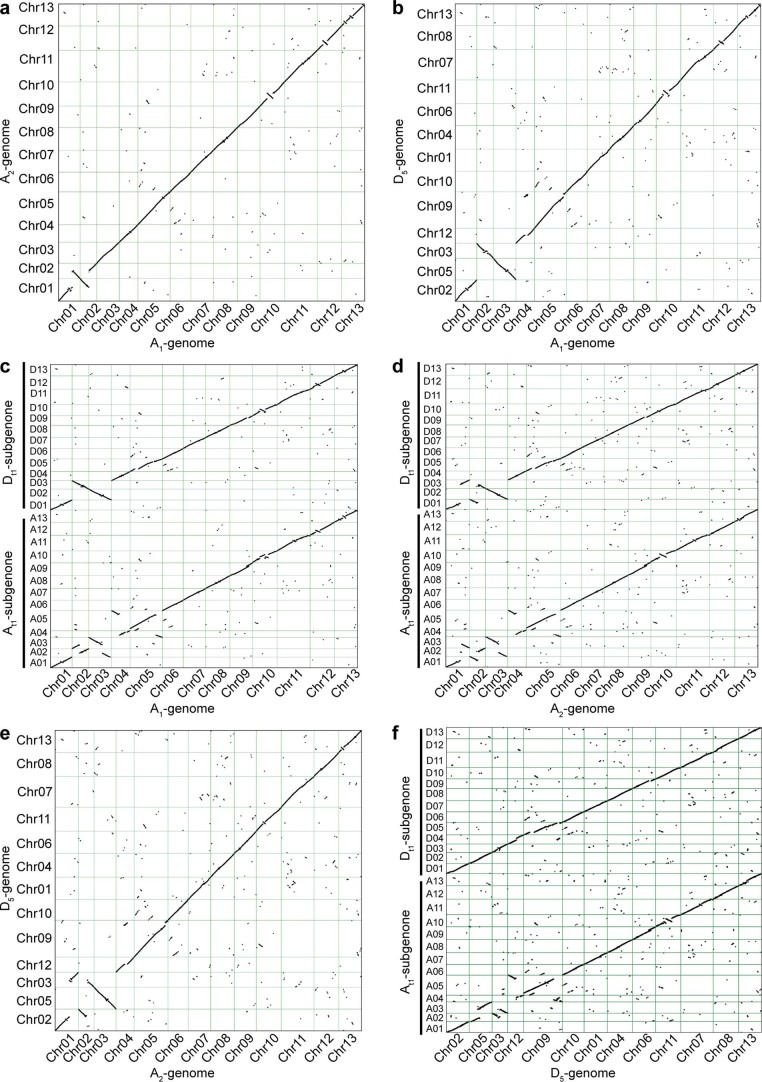
Gene synteny among our assembled A_1_, A_2_, (AD)_1_ genomes and previously released D_5_-genome^16^ sequences. **a**, Dot plot showing gene synteny between A_1_ and A_2_ genomes. **b**, Dot plot showing gene synteny between A_1_ and D_5_ genomes. **c**, Dot plot showing gene synteny between A_1_ and the two subgenomes of (AD)_1_. **d**, Dot plot showing gene synteny between A_2_ and the two subgenomes of (AD)_1_. **e**, Dot plot showing gene synteny between A_2_ and D_5_ genomes. **f**, Dot plot showing gene synteny between D_5_ and two subgenomes of (AD)_1_.

**Extended Data Fig. 3:**
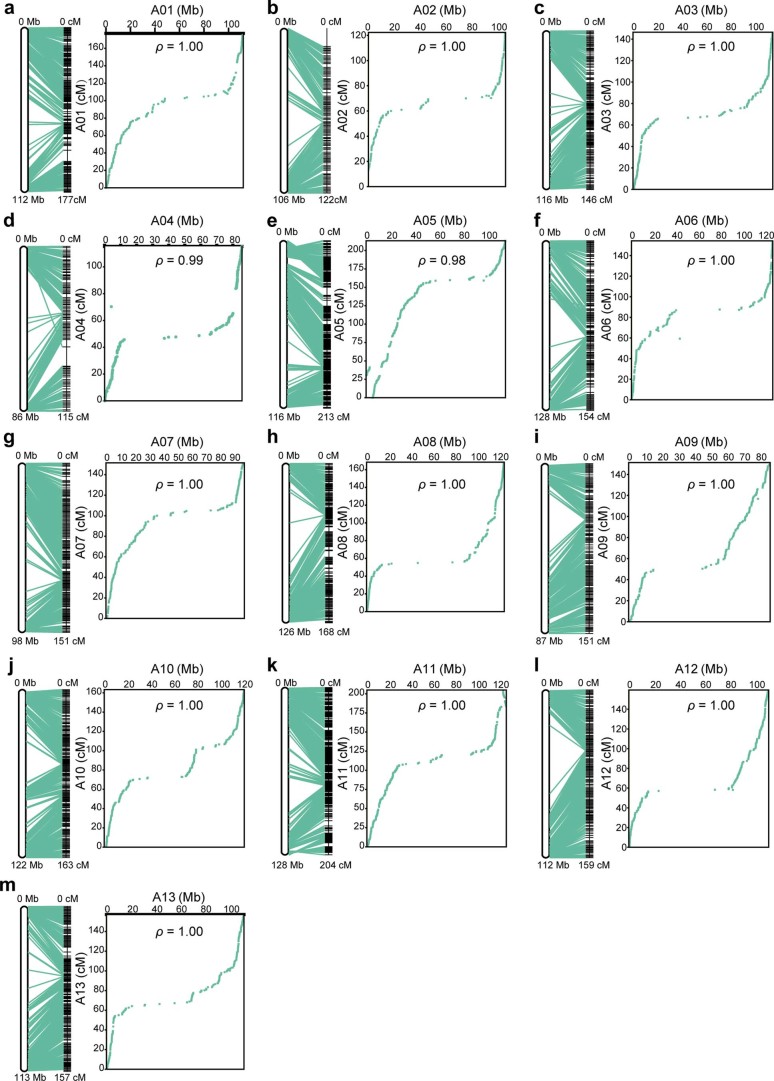
Comparisons of the updated A_t1_-subgenome with a previously reported genetic map. **a**–**m**, Genetic versus physical map distance of the 13 chromosomes of the A_t1_-subgenome in (AD)_1_. A01−A13 (**a** to **m**, respectively), the chromosomes of the A_t1_-subgenome. The *x* and *y* axes represent the physical sequences (in megabases) and genetic distances (in centimorgans), respectively.

**Extended Data Fig. 4:**
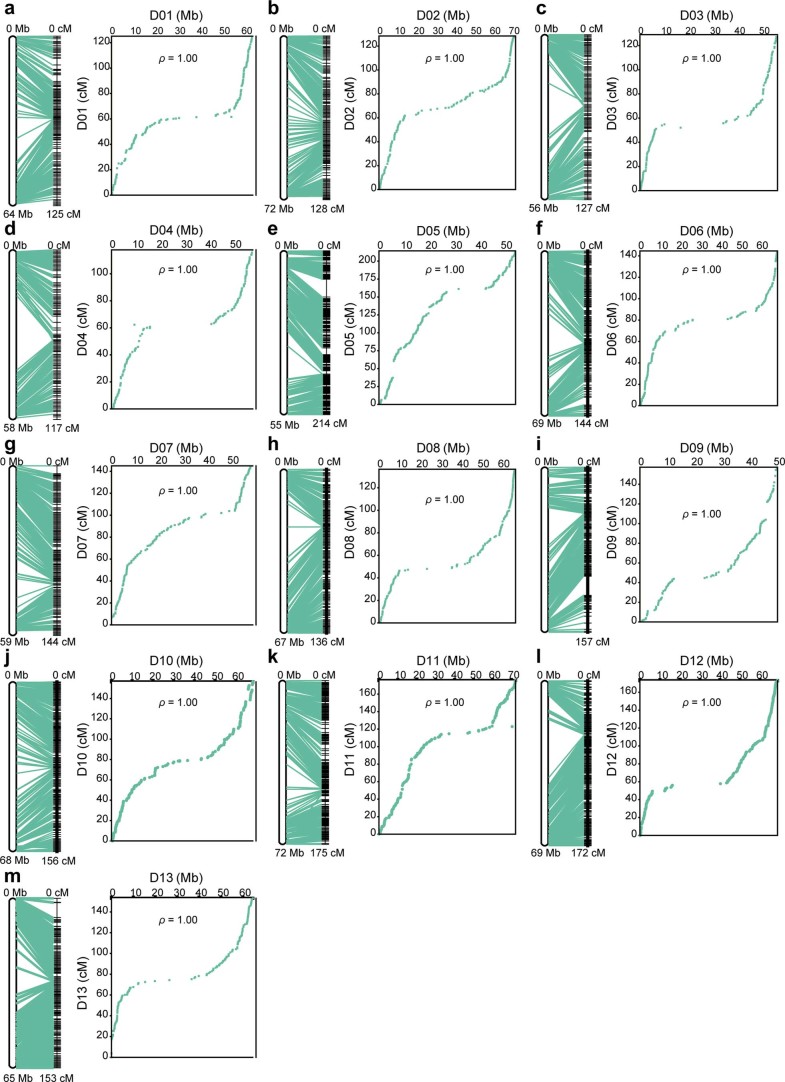
Comparisons of the updated D_t1_-subgenome with a previously reported genetic map. **a**-**m**, Genetic versus physical map distance of the 13 chromosomes of the D_t1_-subgenome in (AD)_1_. D01−D13 (**a** to **m**, respectively), the chromosomes of the D_t1_-subgenome. The *x* and *y* axes represent the physical sequences (in megabases) and genetic distances (in centimorgans), respectively.

Also, the A_2_-genome cultivar Shixiya1 and the (AD)_1_-genome accession TM-1 were further sequenced using high-depth SMRT long reads resulting in 177-fold A_2_-genome coverage (~310 Gb) and 81.6-fold (AD)_1_-genome coverage (~205 Gb), respectively (Supplementary Table 1). The total assembled genome size for A_2_ was 1,637 Mb with 92.18% (1,509 Mb) of all sequences oriented and organized into 13 chromosomes. The resulting assembled genome size for (AD)_1_ was 2,290 Mb with 99.17% of all sequences anchored on 26 chromosomes (A_t1_, 1,449 Mb; D_t1_, 822 Mb). Compared with a recent PacBio-based A_2_ assembly^18^ (8,223 contigs with an N50 of 1,100 kb), our assembly consists of 2,432 contigs with N50 of 1,832 kb, resulting in a reduced number of gaps from 3,707 to 1,163 (Table 1 and Fig. 1a). The N50 of our updated (AD)_1_-genome is 5,020 kb (1,892 kb reported in ref. ^21^), with significantly fewer gaps compared with the most recently published genome (893 gaps versus 2,564 gaps reported in ref. ^21^), which represents ~2.65-fold improvement (Table 1 and Fig. 1b). Our assembled cotton genomes showed high congruence because the strongest signals from the Hi-C data clustered at the expected diagonal (Extended Data Fig. 1). Collinear relationships existed in quantity among cotton genomes, indicating that our pseudo-chromosomes derived from anchored and oriented contigs are of high quality (Extended Data Fig. 2). Our (AD)_1_-genome assembly also shared a high consistency for each chromosome with the previously published genetic map^24^ (Pearson correlation coefficients > 0.98) (Extended Data Figs. 3 and 4). These updated A_2_- and (AD)_1_-genomes may supplant earlier assemblies as chromosome-scale references.

The A_1_-, A_2_- and (AD)_1_-genomes comprise 43,952, 43,278 and 74,350 annotated protein-coding genes (Supplementary Table 2), respectively, mainly in both ends of the chromosomes because as much as 79.71% of A_1_, 80.06% of A_2_ and 64.09% of the (AD)_1_-genome are composed of TEs (Supplementary Table 3 and Fig. 1c). Also, TE-rich regions in the middle region of chromosomes remain silent, with low transcript levels, in contrast to gene-rich regions at both ends of chromosomes with high transcript levels (Fig. 1c).

### Chromosomal translocation and inversions within *Gossypium* lineage

**Extended Data Fig. 5:**
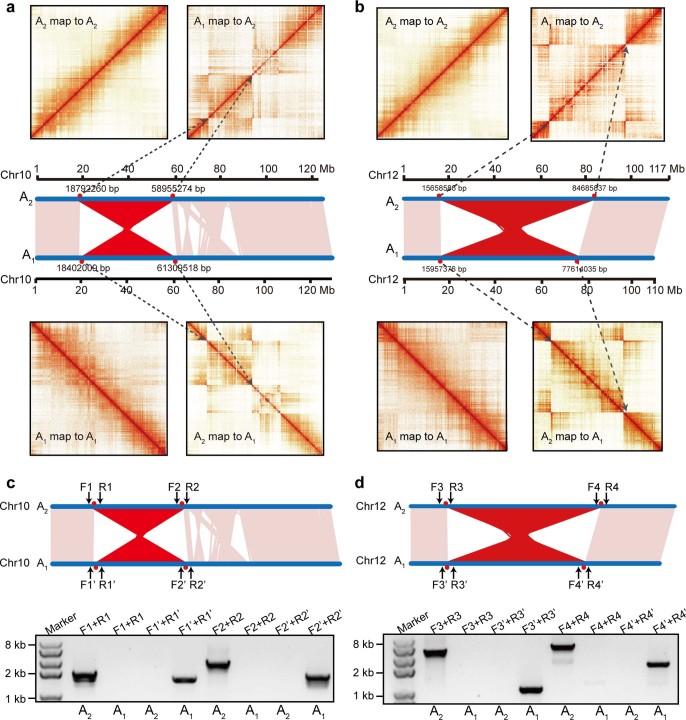
Hi-C data and PCR amplification validate the border of two large inversions in chromosomes 10 and 12 between A_1_ and A_2_ genomes. **a**, **b**, Identification of the ~42.9-Mb large inversion in chromosome 10 (**a**) and ~61.6-Mb large inversion in chromosome 12 (**b**) by Hi-C data. The upper heatmap shows a chromatin interaction matrix that maps Hi-C data from A_2_ against the A_2_-genome (A_2_ map to A_2_), and maps Hi-C data from A_1_ against the A_2_-genome (A_1_ map to A_2_). The middle panel shows a diagram of the inversion region with the four red dots representing the inversion borders. The lower heatmap shows a chromatin interaction matrix that maps Hi-C data from A_1_ against the A_1_-genome (A_1_ map to A_1_) and maps Hi-C data from A_2_ against the A_1_-genome (A_2_ map to A_1_). **c**, **d**, Validation of inversion borders in chromosomes 10 (**c**) and 12 (**d**) by PCR amplification. The forward and reverse primer sequences are shown in Supplementary Table 16. The unprocessed gel for the cropped images are presented in source data. Source data

Compared with that of A_1_, the genome of A_2_ underwent a reciprocal translocation between chromosomes 1 and 2 (Fig. 1d), which is supported by previous cytogenetic data^8^. This translocation likely occurred after the two species separated and then became fixed in A_2_. The A_1_- and A_2_-genomes differed from the A_t1_-subgenome by two and three translocations, respectively, of which the two reciprocal translocations between chromosomes 2 and 3, and 4 and 5, specifically occurred in the tetraploid A_t1_-subgenome (Fig. 1d), suggesting that these translocations probably occurred after polyploidization. The two translocations that specially occurred in A_t1_ were also confirmed by multivalent formations in hybrids between the allotetraploids and A_1_ or A_2_ (ref. ^25^). Two large-scale inversion events were detected between A_1_- and A_2_-genomes in chromosomes 10 and 12 that were confirmed by Hi-C data and also by PCR amplifications (Extended Data Fig. 5). The collinear relationship analysis of these cotton genomes indicated that the inversion in chromosome 12 specifically occurred in A_1_ with the syntenic blocks inverted at the diagonal between ~15.96 Mb and ~77.61 Mb; the inversion in chromosome 10 may have occurred either in A_1_ at the diagonal between ~18.4 Mb and ~61.3 Mb or in A_t1_ between ~23.09 Mb and ~97.42 Mb (Fig. 1e). Thus, the two inversions likely occurred after the speciation of A_1_ and A_2_ cottons.

### Origin of allotetraploid cotton

**Fig. 2:**
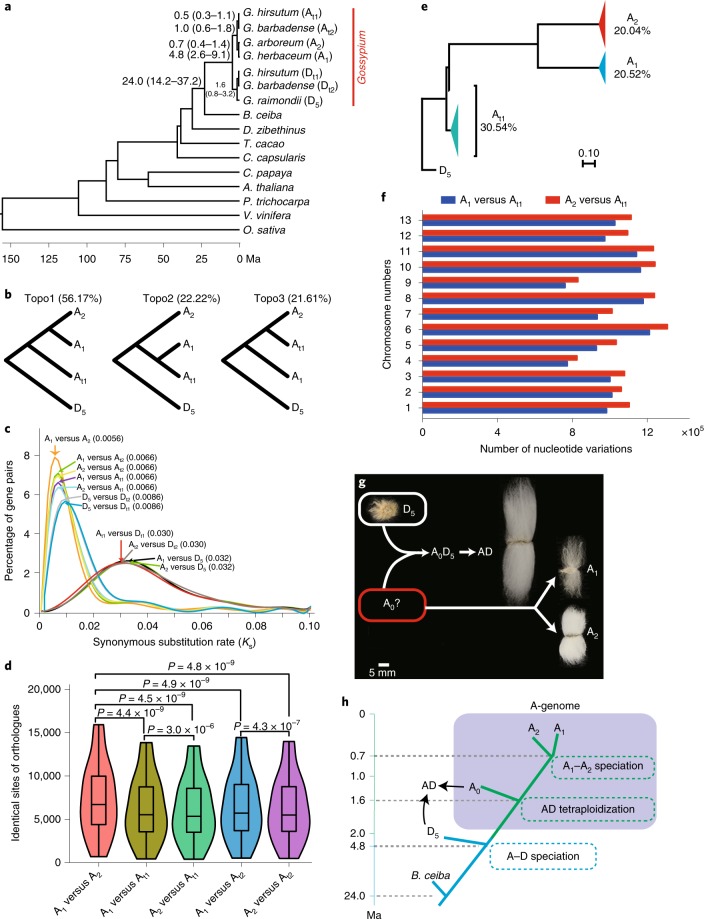
The evolution of the allotetraploid cotton genome. **a**, Inferred phylogenetic analysis among *Gossypium* and other eudicot plants. **b**, Summary of phylogenetic analysis with the approximately unbiased test in 10-kb windows. **c**, Distribution of *K*_s_ values for orthologous genes among cotton genomes. Peak values for each comparison are indicated in the parentheses. **d**, Comparisons of identical sites in orthologous genes. Violin plots summarize the distribution of identical sites. The center line in each box indicates the median, and the box limits indicate the upper and lower quartiles of divergence (*n* = 20 types of synonymous mutation). *P* values were derived with Student’s *t*-test. **e**, Phylogenetic and ancestral allele analysis based on SNPs. The red, blue and green triangles represent the collapsed 21 A_2_ accessions, 14 A_1_ accessions and 30 (AD)_1_ accessions, respectively. The percentage value indicates the percentage of ancestral alleles for each species that were identical to those of the D_5_-genome. **f**, Number of nucleotide variations in A_1_ or A_2_ compared with A_t1_ across the chromosomes. **g**, A model for the formation of allotetraploid cotton showing fiber phenotypes from the (AD)_1_ (accession TM-1), the D_5_, the A_1_ (var. *africanum*) and the A_2_ (cv. Shixiya1). Scale bar, 5 mm. **h**, A schematic map of the evolution of cotton genomes. Major evolutionary events are shown in dashed boxes. Source data

**Extended Data Fig. 6:**
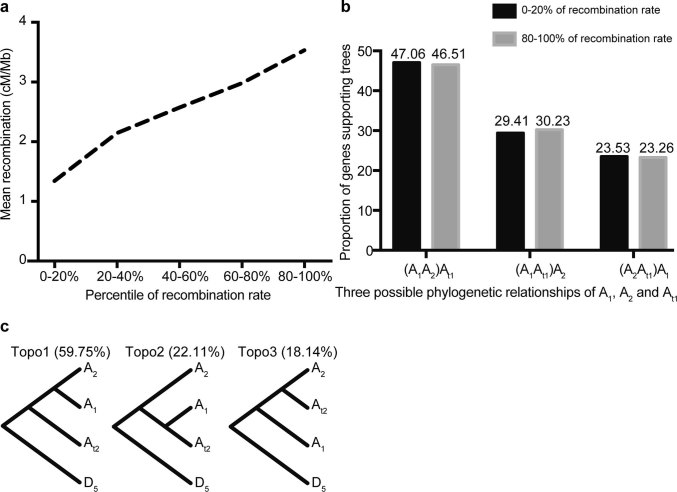
Phylogenetic relationship among A_1_, A_2_ and A-subgenome. **a**, Distribution of mean recombination rates in protein-coding genes with available recombination rates (n = 240 genes). **b**, Proportion of genes with 0-20% and 80-100% of recombination rates supporting the three trees. **c**, Summary of phylogenetic analysis with the AU test in 10 kb windows among A_1_, A_2_, A_t2_, D_5_.

**Extended Data Fig. 7:**
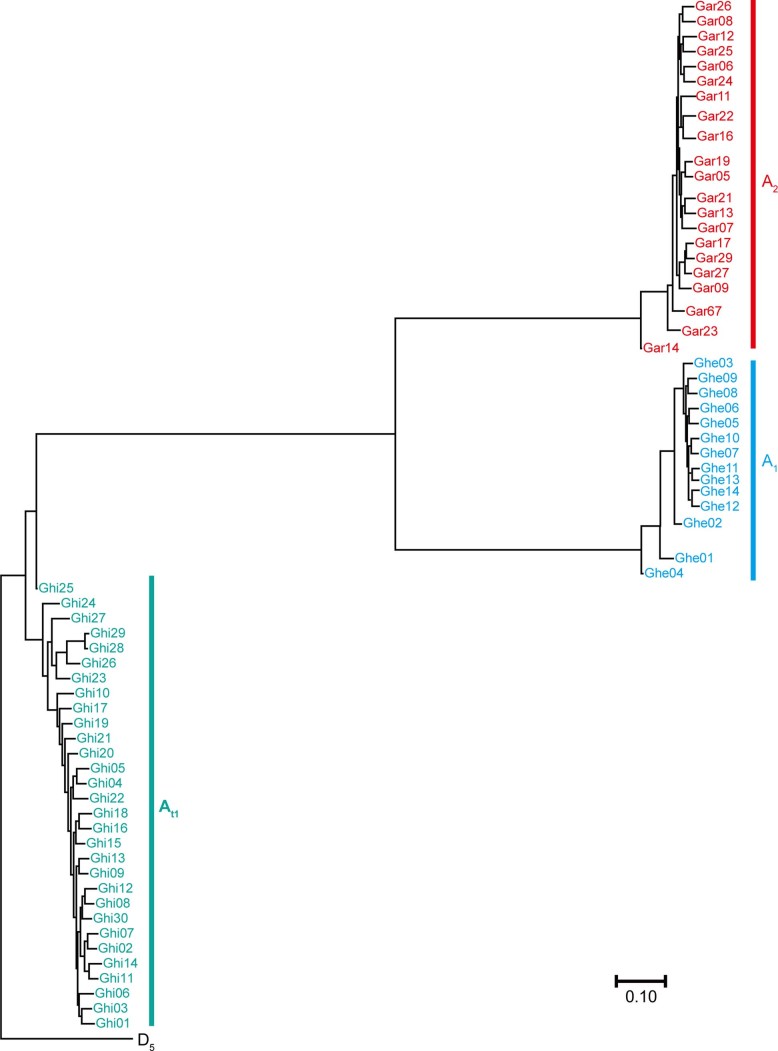
A phylogenetic tree based on SNPs in the genomes with D_5_-genome as the outgroup. Units (as measured by the indicated scale) show the percentage of represented polymorphic sites that differed between two individuals. Detail information of cotton accessions were described in Supplementary Table 4.

A molecular tree based on single-copy genes suggests that the common ancestor of the A_1_ and A_2_ clade was phylogenetically a sister to the A_t_-subgenomes (A_t1_ and A_t2_) of (AD)_1_ and of (AD)_2_, respectively, and the divergence time for A_1_ and A_2_ was estimated to be ~0.7 Ma (0.4−1.4 Ma), well after the allotetraploid formation ~1.0−1.6 Ma (the values for the separation of A_t_ to A_1_ or A_2_, and D_t_ to D_5_) (Fig. 2a). Gene trees with specific recombination regions also supported the sister relationships between the A_1_–A_2_ clade and A_t1_ (Extended Data Fig. 6a,b). Whole-genome phylogenetic analysis showed that the major topology 1 (A_t1_, 56.17%; A_t2_, 59.75%) supported the constructed species tree in Fig. 2a. The minor topology 2 with the sister relationship of A_1_ and A_t_ (A_t1_, 22.22%; A_t2_, 22.11%) had a slightly higher rate than the other minor topology 3 with the sister relationship of A_2_ and A_t_ (A_t1_, 21.61%; A_t2_, 18.14%) (Fig. 2b and Extended Data Fig. 6c). Synonymous substitution (*K*_s_) analysis indicated that A_1_ and A_2_ had the lowest divergence (*K*_s_ values), compared with all other pairs (Fig. 2c). Likewise, a significantly greater number of identical sites were found between orthologs of A_1_ versus A_2_ relative to either A_1_ or A_2_ versus A_t1_ or A_t2_ (Fig. 2d). We further selected representative cotton lines, including 30 (AD)_1_, 14 A_1_ and 21 A_2_ accessions, to construct a phylogenetic tree based on whole-genome SNP studies to further validate the relationships of A_1_, A_2_ and A_t1_ (Fig. 2e and Extended Data Fig. 7). Because the actual A-genome donor may be extinct, we compared A_t1_, A_1_ and A_2_ accessions with the D_5_, an outgroup for all A-genome species. The distance from D_5_ to A_t1_ was much smaller than that from D_5_ to its previously thought common ancestor, A_1_ or A_2_. About 30.54% of the SNPs of A_t1_ were identical to the corresponding sites in the D_5_-genome, whereas only 20.52% and 20.04% of ancestral alleles of A_1_ and A_2_, respectively, were identical to the corresponding sites in D_5_-genome (Fig. 2e). The nucleotide variation analysis indicated that A_1_ has relatively fewer nucleotide variations than A_2_ compared with A_t1_ across the 13 chromosomes (Fig. 2f). Based on these evidence, we constructed a revised model in which neither A_1_ nor A_2_ is the actual A-genome donor. Instead, hybridization between the common ancestor (A_0_) of all A-genomes (A_1_, A_2_ and A_t_) and a D_5_-genome resembling *G. raimondii* formed the allotetraploid cotton (Fig. 2g). Our results also indicated that the A_0_, inferred as the possible A_t_ donor, was more phylogenetically related to A_1_ than A_2_. The AD ((AD)_1_ and (AD)_2_) tetraploidization occurred approximately 1.0−1.6 Ma; A_0_ then developed into two A-genomes around 0.7 Ma (Fig. 2h). The finding that A_0_ is a common ancestor for A_1_, A_2_, the A_t1_-subgenome in (AD)_1_ and the A_t2_-subgenome in (AD)_2_ resolves a puzzle regarding previous inconsistent phylogenetic data^6–9,11–13^ and explains why interspecific hybridization of A_1_ or A_2_ with D_5_ is often unsuccessful, because the genetic distances between the current A- and D-genomes are great enough to preclude fertilization.

### Population genomic study of two A-genome species

**Fig. 3:**
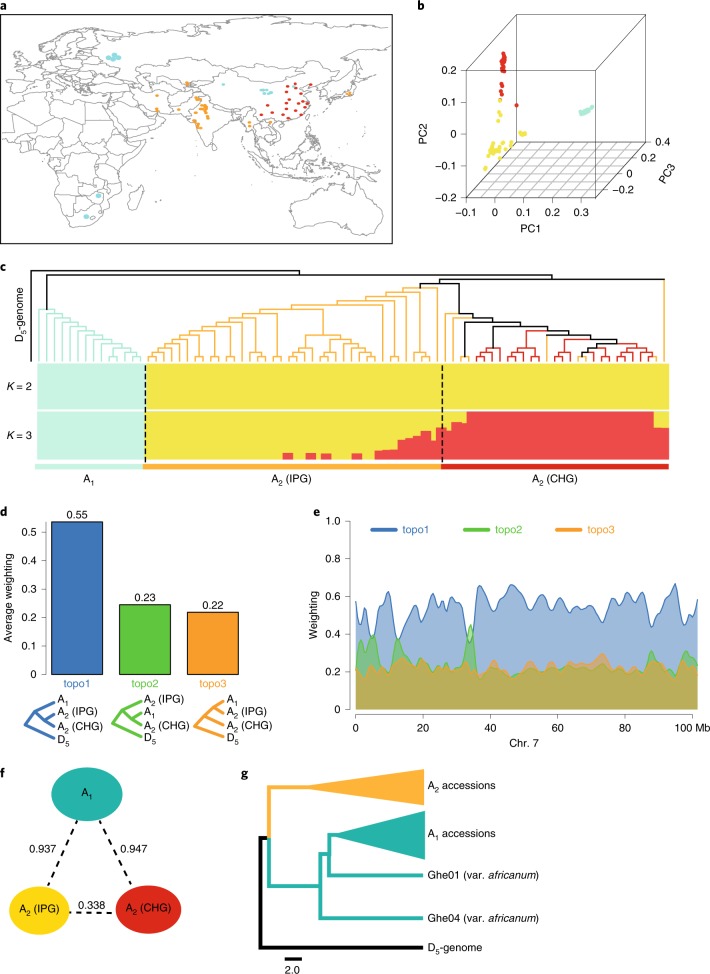
Geographic distribution and population analysis of the A_1_ and A_2_ accessions. **a**, Geographic distribution of the collected A_1_ and A_2_ accessions. Green, red and yellow dots represent A_1_ accessions and A_2_ accessions collected in China and outside of China, respectively. The map was drawn using the maptools package (http://maptools.r-forge.r-project.org/). **b**, PCA plots of the first three components for A_1_ and A_2_ accessions. Dot colors are the same as in **a**. **c**, Analysis of genetic relationship between all A_1_ and A_2_ accessions. The upper and lower panels show the phylogenetic tree based on whole-genome SNP studies and population structure of all accessions based on different numbers of clusters (*K* = 2–3), respectively. Branch colors are the same as in **a**. CHG, A_2_ accessions from the China group; IPG, A_2_ accessions from the India and Pakistan group. **d**, Average weightings for the three possible topologies in whole genomes. **e**, Weightings for all three topologies described in **d** across chromosome 7 using sliding windows. **f**, Population divergence (*F*_ST_) across the three groups described in **c**. **g**, Phylogenetic analysis based on SNPs. The yellow and green triangles represent the collapsed 67 A_2_ accessions and 12 A_1_ accessions, respectively. Two A_1_ var. *africanum* accessions (Ghe01 and Ghe04) gathered at the root of the 12 A_1_ accessions. PC1, the first principal component (PC); PC2, the second PC; PC3, the third PC.

**Extended Data Fig. 8:**
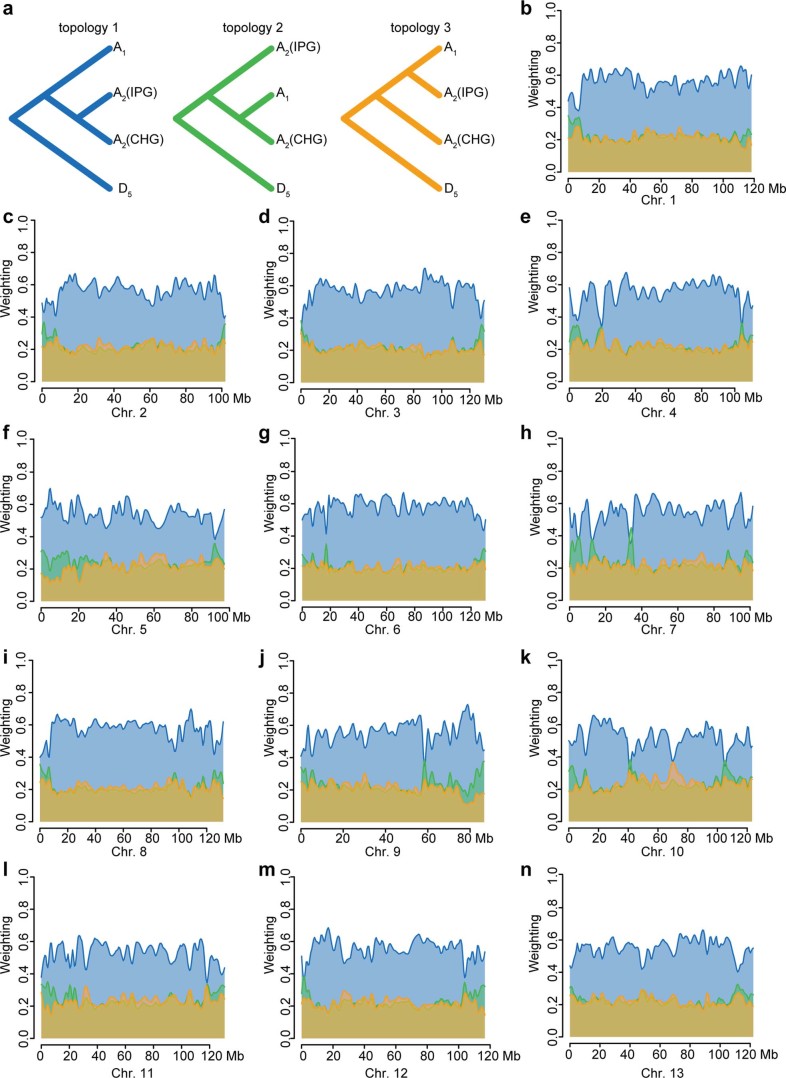
Topology weighting for A_1_ and A_2_ populations. **a**, Three possible taxon topologies for A_1_, A_2_ from China group (CHG) and A_2_ mostly from India and Pakistan group (IPG) with D_5_ as the outgroup. **b-n**, Weightings for all three topologies plotted across the 13 chromosomes. Colour for each topology corresponds to coloring in **a**.

We collected 14 A_1_ and 67 A_2_ representative cotton accessions from India, Pakistan, China and other countries to study the genetic divergence between A_1_ and A_2_ (Fig. 3a and Supplementary Table 4). All resequencing reads with an average coverage depth of ~7.2× for each accession were mapped to our assembled A_2_-genome for SNP identification. A total of 11,652,404 SNPs and 1,716,908 indels (ranging from 1 to 259 base pairs (bp) in length) were identified (Supplementary Table 5). Principal component analysis (PCA) based on SNPs showed that, despite their geographic origins, these cotton accessions were clustered in two independent groups: the A_1_ group and A_2_ group (Fig. 3b). The neighbor-joining tree using SNPs indicated that A_1_ and A_2_ clustered in two independent clades, and A_2_ from India and Pakistan and A_2_ from China have the closest relatives (Fig. 3c), which was confirmed by sliding window phylogenetic analysis with an average weighting of 55% in topology 1 (Fig. 3d). The topology 2 is nearly identical to topology 3 throughout chromosomes, but there are several weak shifts in support toward topology 2 potentially reflecting the introgression between A_1_ and A_2_ distributed in China (Fig. 3e and Extended Data Fig. 8). Model-based clustering showed that the population structures of A_1_ accessions were obviously significantly different from A_2_ accessions (number of clusters (*K*) = 2), and the population divergence between the A_1_ and A_2_ from the India and Pakistan group or from China reached almost 1.0, which suggested that these differences clearly distinguish A_2_ from A_1_ as two cotton species, and may explain the phenomenon in which interspecific hybridizations of A_2_ with A_1_ are often unsuccessful (Fig. 3c,f). Several A_2_ accessions from India and Pakistan were clustered sisterly to all A_2_ accessions collected from China, and the accessions from China had distinct population structures from accessions from India and Pakistan (*K* = 3). According to our results and the recorded history of Chinese Asian cotton^26^, we concluded that A_2_ was likely introduced to China from India and/or Pakistan, then developed into a distinct geographical race (Fig. 3c). Two accessions of A_1_ var. *africanum* were gathered at the root of all other A_1_ accessions with no obvious impact on A_2_ development, which did not support the notion that *africanum* is the source of both cultivated A_1_- and A_2_-genomes^14^ (Fig. 3g). The large genetic differences revealed by population analysis and chromosomal SVs between A_1_ and A_2_ suggest that two A-genomes were evolved independently, with A_1_ var. *africanum* as the only living ancestor of A_1_ accessions.

### Genome expansions and evolution

**Fig. 4:**
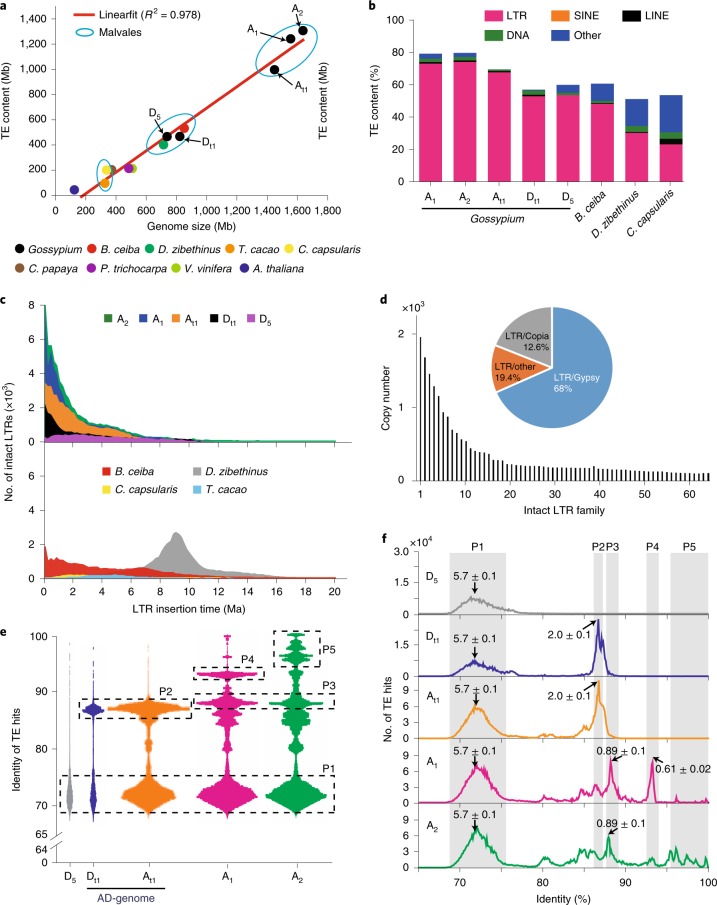
Genome expansions in sequenced Malvales plants, particularly in cotton, and quantitative and comprehensive analysis of LTRs, especially Gypsy-type. **a**, Genome size expansion is highly correlated with TE amplification bursts (*R*^2^ = 0.978). The red line shows the linear relationship between genome size and TE content. **b**, Genomic component comparisons among genome-sequenced Malvales plants. **c**, Analysis of intact LTR numbers and insertion time in Malvales plants. **d**, Classification of intact LTRs in the A_2_-genome. LTR families with a copy number of ≥100 are shown. **e**, Identity distribution pattern of TE hits presented as a dot-plot. The most recent LTR/Gypsy sequence of LTR families was selected as the representative sequence for detecting additional TE hits in the genomes. A total of 262,377 dots in D_5_, 585,658 in D_t1_, 3,541,372 in A_t1_, 4,218,810 in A_1_ and 5,035,006 in A_2_ were drawn in the dot-plot. P1–P5 represent the identified five distinct bursts in different cotton genomes. **f**, Number of TE hits for the representative sequence and their associated identity values. The estimated burst time based on GPDF fitting of each peak is marked. The five peaks, P1–P5, defined in **e** are highlighted by shaded gray columns. LINE, long interspersed nuclear elements; SINE, short interspersed nuclear elements.

**Extended Data Fig. 9:**
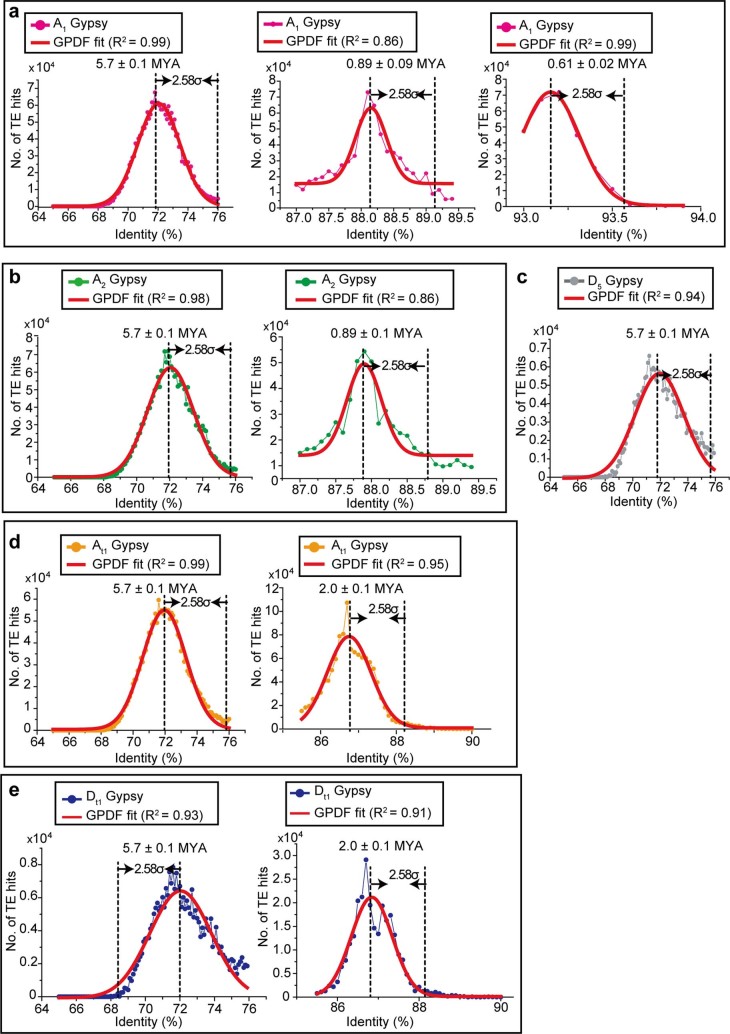
The burst of LTR/Gypsy amplification fits a Gaussian distribution and allows time estimation of burst events. **a**-**e**, GPDF modeling data fit well with the actual TE bursts found in A_1_ with R^2^ from 0.86–0.99 (**a**), A_2_ with R^2^ from 0.86–0.98 (**b**), D_5_ with R^2^=0.94 (**c**), A_t1_ with R^2^ from 0.95–0.99 (**d**) and D_t1_ with R^2^ from 0.91–0.93 (**e**). The estimated time of the LTR/Gypsy burst event associated with each genome or subgenome is shown in the graphs. The peak width was defined as 2.58σ that covers >99.5% of the nucleotide substitution events in TEs.

Among genome-sequenced plants of the order Malvales^27–30^, D_5_ and the D_t1_-subgenome in (AD)_1_ are similar in genome sizes relative to *Bombax ceiba* or *Durio zibethinus*, but are expanded nearly twofold compared with the *Theobroma cacao* and *Corchorus capsularis* genomes (Fig. 4a). The two A-genomes and the A_t1_-subgenome experienced a further twofold expansion that was highly correlated with TE bursts (correlation coefficient, *R*^2^ = 0.978) (Fig. 4a). While both the D_5_-genome (738 Mb) and D_t1_-subgenome (822 Mb) are nearly equivalent in size relative to the *D. zibethinus* genome (715 Mb), long terminal repeat (LTR) families in *Gossypium* (52.42% of the D_t1_-subgenome, 53.2% of the D_5_-genome) were greatly expanded in comparison to *D. zibethinus* (26.2%). As much as 72.57% of the A_1_-genome and 73.62% of the A_2_-genome were composed of LTRs (Fig. 4b). LTR retrotransposons in *Gossypium* and *B. ceiba* have experienced continuing and more recent amplification bursts from 0−2 Ma, while *D. zibethinus* underwent a distinct amplification burst event around 8−10 Ma (Fig. 4c). LTR retrotransposons in the A_2_-genome were further classified into 64 families, of which 68% belonged to the Gypsy superfamily and 12.6% to Copia (Fig. 4d). By using representative LTR/Gypsy sequences (Supplementary Fig. 1) to evaluate TE hits in cotton genomes, five distinct insertion peaks for the Gypsy-type LTR with identities from 65−76% to 96.4−99.4% were observed in different cotton genomes (Fig. 4e). We used our Gaussian probability density function (GPDF) analysis to estimate the burst time of major peaks, finding that the earliest insertion event occurred ~5.7 Ma, which is the expected speciation time for A- and D-genomes (Extended Data Fig. 9 and Fig. 4f). The peak with 85.5−88.5% identity, corresponding to ~2.0 Ma, is found specifically in D_t1_- and A_t1_-genomes, but not in D_5_, A_1_ or A_2_, suggesting that the allotetraploid cotton may have formed as early as ~2.0 Ma. The peak with 87−89.5% identity corresponded to 0.89 Ma and is common to both A_1_ and A_2_, indicating that speciation might occur at a later time. Indeed, the 93.0−93.8% identity (or 0.61 Ma) peak is unique to A_1_, and the last peak (with 96.4−99.4% identity; no valid calculation of ages because it is too close to date) is A_2_-specific. Our data showed that A_1_ and A_2_ speciation occurred 0.89−0.61 Ma. This was confirmed by results (Supplementary Fig. 2a–c, TDIV1 (divergence time between A_1_ and A_2_) = 1,016,499 yr) obtained from *fastsimocoal2* analysis, which used 30 accessions from (AD)_1_, 14 from A_1_ and 21 from A_2_, as reported in Fig. 2e. However, G-PhoCS analysis, which used data from the fully assembled A_1_-, A_2_- and (AD)_1_-genomes (Supplementary Fig. 2d–f), did not quite fit our previous model. We suggest that G-PhoCS may not fit well for evolutionary analysis of genomes with high TE contents, such as cotton.

### SVs and fiber development

**Fig. 5:**
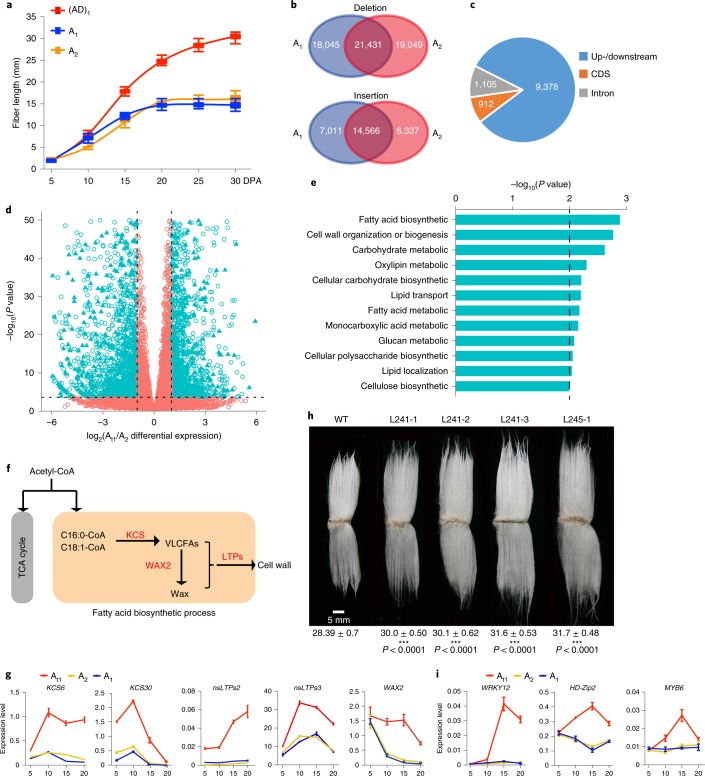
SV analysis among A_t1_, A_1_ and A_2_. **a**, Comparisons of fiber elongation patterns. The center line in each box indicates the median, and the box limits indicate the upper and lower quartiles (*n* = 30 seeds). **b**, SVs of two A-genomes compared with the A_t1_-subgenome. **c**, Annotation of identified common SVs in genic regions. Up-/downstream, 5 kb regions from the start or stop codons. **d**, Volcano plots for A_2_~A_t1_ gene expression in elongating fibers at 15 DPA. Each hollow point represents a gene and genes with SVs within 5 kb of their start or stop codons are indicated by a triangle. Dashed lines show the thresholds (*P* ≤ 0.001 and twofold change between A_2_ and A_t1_). **e**, Gene ontology enrichment of significant differentially expressed genes with SVs (*P* ≤ 0.01). **f**, Upregulated genes in fatty acid biosynthetic process. Red items, upregulated genes in A_t1_ relative to A_2_ at 15 DPA. **g**, RT–qPCR analysis of upregulated genes in fatty acid biosynthetic pathway in elongating fibers at 5–20 DPA. *UBQ7* was used as a normalization control (mean ± s.d, *n* = 3 independent experiments). **h**, Cotton fibers of the WT (*G. hirsutum* cv. Zhong24) and the transgenic lines expressing *KCS6* gene under control of the CaMV 35S promoter (L241-1, L241-2 and L241-3) or E6 promoter (L245-1). The averaged fiber lengths with standard errors are denoted under each cotton line using Student’s *t*-test. Scale bar, 5 mm. **i**, RT–qPCR analysis of three upregulated potential transcription factor genes in elongating fibers at 5–20 DPA (mean ± s.d., *n* = 3 independent experiments). WT, wild type. Source data

**Extended Data Fig. 10:**
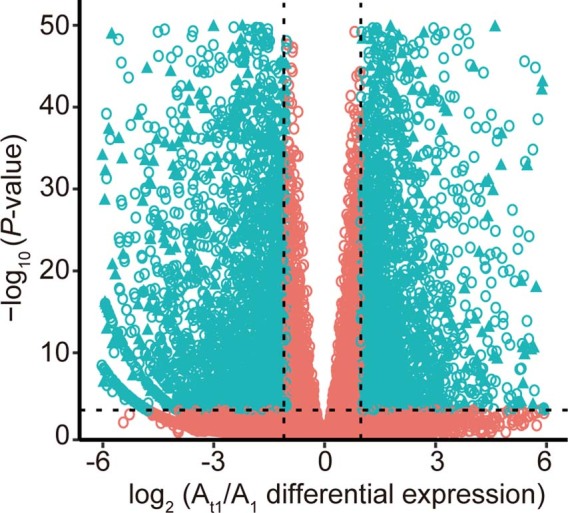
Volcano plots for A_1_-A_t1_ gene expression in elongating fibers at 15 DPA. Each hollow point represents a gene and genes with SVs within 5 kb of their start or stop codons are indicated by a triangle. The dashed lines show the thresholds (*P*-value ≤ 0.001 and two-fold change).

SVs including large deletions and insertions (>50 bp) are reported to drive important phenotypic variation within species^31^. Here we found that (AD)_1_ fiber cells underwent fast elongation reaching up to 30.5 ± 0.7 mm until 30 d post anthesis (DPA), whereas fiber cells in A_1_ (14.7 ± 0.7 mm) and A_2_ (16.1 ± 0.9 mm) elongated at a slower rate and terminated earlier (~20 DPA) (Fig. 5a). By comparing two A-genomes with the A-subgenome of (AD)_1_, we identified 39,476 deletion and 21,577 insertion events in A_1_, as well as 40,480 deletion and 20,903 insertion events in A_2_. Meanwhile, we obtained 35,997 common SVs events including 21,431 deletions and 14,566 insertions in A_1_ and A_2_, suggesting that these SVs occurred mainly at the common ancestor stage of two A-genome species (Fig. 5b). Of the total common SVs, 11,395 events (31.66%) were overlapped with genic regions affecting 9,839 unique genes, with 912 events occurring in coding DNA sequences (CDSs), 1,105 in introns and 9,378 in up-/downstream regions (Fig. 5c and Supplementary Table 6). Of the reported 1,753 associated loci for fiber traits^2,32^, 460 associated loci contained common SVs, with those in up-/downstream regions as the major type (Supplementary Table 7). We identified 1,545 upregulated and 1,908 downregulated genes by comparing transcriptomes of rapidly elongating fiber cells from the A_t1_-subgenome with those of A_2_ (Supplementary Table 8). Also, 2,941 upregulated and 3,350 downregulated genes were identified with A_t1_ and A_1_ comparisons at elongating fibers (Supplementary Table 9). Of these differentially expressed genes, 949 for A_t1_ versus A_2_ and 1,687 for A_t1_ versus A_1_ contained common SVs, respectively (Fig. 5d, Extended Data Fig. 10 and Supplementary Tables 10 and 11). Gene ontology enrichment analysis indicated that fatty acid biosynthesis, cell wall deposition or biogenesis, and carbohydrate metabolism were the most enriched biological processes (Fig. 5e). Quantitative PCR with reverse transcription (RT–qPCR) analysis of several key genes related to fatty acid biosynthesis, including encoding 3-ketoacyl-CoA synthase (KCS), fatty acid hydroxylase (WAX2) and lipid transport proteins, validated the upregulation pattern in A_t1_ compared with both A_1_ and A_2_ (Fig. 5f,g). Large sequence variations existed between A_t1_ and A_1_ or A_t1_ and A_2_ in the upstream or downstream regions of all of these genes (Supplementary Fig. 3). We introduced *KCS6*, a key gene in very-long-chain fatty acid biosynthesis^33,34^, in *G. hirsutum* cv. Zhong24 background and observed a significant increase (~6.0–11.66%) of final fiber lengths in three homozygous transgenic lines (L241-1, L241-2, L241-3) that were driven by 35S promoter and one line (L245-1) driven by the fiber-specific E6 promoter (Fig. 5h). Fifty-six transcription factors, including WRKY12, HD-Zip2 and MYB6, showed differential expression patterns among the three cotton species that can be correlated with SVs (Fig. 5i and Supplementary Table 12). In combination with genome scanning of transcription factor binding sites and A_2_–A_t1_ differential expression, we identified 198 potential target genes for WRKY12 and 232 for HD-Zip2 in the cotton genome (Supplementary Tables 13 and 14). We suggest that higher expression intensities of these potential target genes in (AD)_1_ may lead to longer fibers in (AD)_1_ than in either A_1_ or A_2_.

## Discussion

With high-quality assembly of two African–Asian species, A_1_ and A_2_, we provided a more complete landscape of genome architecture, gene annotations and TE insertions, which is critical to evolutionary and comparative genomics as well as genetic variation analysis. Our data suggested that A_t_ may have originated from a primitive A-genome common ancestor, referred to here as A_0_, instead of extant A_1_ or A_2_. Allotetraploid formation preceded the speciation of the present two A-genomes, and then A_1_ and A_2_ originated independently with no ancestor–progeny relations. Upon publication of our new data, we anticipate that reviews and textbooks^7,11,35^ related to cotton genome evolution will have to be revisited and revised.

Several LTR bursts contributed compellingly to A-genome size expansion, speciation and evolution. By using fragmented coding sequences of LTRs, our GPDF analysis overcame a major pitfall related to most previous similar studies that relied on the presence of both ends of full-length LTRs^10,17,36,37^, such that more recently inserted LTRs are likely over-represented. We suggest that GPDF may be applied to analyze accurately the time of LTR bursts and genome evolution. Analysis of SV and gene expression patterns identified putative candidates to investigate the phenotypic difference among three cotton species. These candidate genes would enable cotton breeders to further improve major agronomical traits such as fiber quality and yield.

## Methods

### Sampling and sequencing

Genomic DNA molecules of *G. herbaceum* (var. *africanum* Mutema, A1-0076), *G*. *arboreum* (cv. Shixiya1) and *G. hir*s*utum* (TM-1) were isolated from young leaves of individual plants. We obtained polymerase reads of ~225 Gb, ~177 Gb and ~205 Gb from SMRT cells on PacBio RSII and Sequel instruments for *G. herbaceum* (A_1_), *G*. *arboreum* (A_2_) and *G. hir*s*utum* (AD)_1_, respectively. Our previously released ~133-Gb PacBio reads from A_2_ were also integrated into our current A_2_-genome assembly. For A_1_-, A_2_- and (AD)_1_-genomes, we also obtained ~52 Gb, 95 Gb and 70 Gb of raw reads, respectively, with 400-bp inserts using a whole-genome shotgun approach on the Illumina HiSeq X-Ten platform. We sequenced ~256 Gb of clean Hi-C data for A_1_, ~219 Gb of clean Hi-C data for A_2_ and ~196 Gb of clean Hi-C data for (AD)_1_ on the Illumina HiSeq platform.

### Assembly and correction

We performed de novo assembly of PacBio long reads into contigs with the program Falcon (v.0.4)^38^. To further improve the accuracy of reference assembled contigs, two-step polishing strategies were performed: we first used PacBio long reads and carried out an initial polishing with Polish software^39^ and then used highly accurate Illumina paired-end reads to further correct the assembly with Pilon (v.1.20) software^40^. The PacBio contigs were further clustered and extended into pseudo-chromosomes using Hi-C data. Gaps that existed in the genomes were filled using Pbjelly^41^, followed by a second round of polishing using Quiver^39^.

### Repeat analysis

Each of the whole genomes was searched for repetitive sequences including tandem repeats and TEs. Tandem repeats were annotated by TRF (v.4.07b)^42^ with the following parameters: 2, 7, 7, 80, 10, 50, 2,000. TE annotations were identified using a combination of de novo and homology-based approaches. A de novo repeat library was constructed with RepeatModeler (v.1.0.8). We adapted RepeatMasker (v.4.0.6)^43^ to search for similar TEs against Repbase (Repbase21.08)^44^, mips-REdat library and the de novo repeat library. The RepeatProteinMask program was used to search against a TE protein database.

### Analysis of potential LTR bursts using fragmented Gypsy-type transposons derived from full-length sequences

Intact LTR retrotransposons were detected using LTR_FINDER^45^ and classified into 64 families with 5′-LTR sequences based on the following parameters: similarity ≥ 80%, coverage ≥ 80% and copy number ≥ 100. A total of 13,332 LTR retrotransposons were translated in six frames that produced 1,397 Gypsy sequences with amino acids > 1,000.

### GPDF fitting of LTR identity distributions and LTR burst time calculations

Full-length and truncated LTRs were identified across genomes with various lengths and identities, and then each sequence (length = *l*) was divided into 30-bp units to determine the number of dots (*n* = *l*/30) with the same identity. Each Gypsy superfamily sequence was normalized to dot arrays with various identities, and all dot arrays were used to generate a box-plot according to their identities. For GPDF fitting and burst time calculation, single peaks in the TEs identity distribution curves were separated and fitted by GPDF with high adjusted *R*^2^ values, and the average nucleotide substitution ratio (*K*) was defined as 2.58 standard deviations (*σ*). Then the TE burst time point for individual amplification peaks was estimated by *t* = *K*/*r*, in which *r* is the nucleotide substitution rate for cotton species (*r* = 7 × 10^−9^)^17^.

### Gene prediction and annotation

Homology-based prediction, RNA-sequencing-assisted prediction and ab initio prediction were used for gene model prediction. For homology-based prediction, GeMoMa software^46^ was applied based on homologous proteins from sequenced species, which included *Arabidopsis thaliana* (TAIR10, http://www.arabidopsis.org/), *Oryza sativa* (v7.0), *G*. *arboreum*, *G*. *hirsutum*, *G*. *raimondii* (D_5_), *Populus trichocarpa* (v.3.1), *T. cacao* (http://cocoa-genome-hub.southgreen.fr) and *Vitis vinifera* (Genoscope 12×). RNA sequencing transcripts assembled with HISAT^47^ and StringTie^48^ were used to assist in gene structure predictions (Supplementary Table 15). In summary, a total of 52,444 (mean size: 2,177.9 bp), 56,130 (mean size: 2,414.7 bp) and 111,872 (mean size: 1,892.1 bp) assembled transcripts were obtained for A_1_, A_2_ and (AD)_1_, respectively. For ab initio gene prediction, we applied SNAP (V2006-07-28)^49^, Augustus (v.3.2.2)^50^, Genscan^51^ and GlimmerHMM (v.3.0.4)^52^ to generate gene structures. Finally, all predictions were integrated to produce a consensus gene set using EVidenceModeler (v.1.1.1)^53^. Gene functional annotations were assigned by aligning protein sequences to Swiss-Prot and TrEMB^54^ using BLASTP (*E* value (expected value) ≤ 1 × 10^−5^), KAAS^55^ (v.2.1) and InterProScan^56^ (v.5.24). Gene Ontology^57^ IDs for each gene were extracted from the InterPro entry.

### Phylogenetic analysis

We used BLASTP to generate protein sequence pairs (*E* value ≤ 1 × 10^−5^) and then OrthoMCL (v.2.0.9)^58^ to cluster gene families with an inflation value of 1.5. The single-copy gene families were extracted and aligned using MAFFT (v.7.058)^59^. A phylogenetic tree was constructed using a maximum likelihood method implemented in RAxML (v.8.0.19)^60^ with a GTRGAMMA substitution model with *O. sativa* as the outgroup. The Markov chain Monte Carlo algorithm for Bayes estimation was adopted to calculate the divergence time using PAML (v.4.6)^61^. For the identification of SNPs on orthologous genes among A_1_, A_2_, A_t1_ in (AD)_1_ and A_t2_ in (AD)_2_ (ref. ^21^), we used BLASTP to do pairwise alignments and retained only homologous gene pairs with reciprocal best hits (*E* value ≤ 1 × 10^−5^). Then we generated multiple alignments of homologous proteins and back-translated to the CDS. A SNP was determined to be present if a position in the alignment included two or more different bases. If a SNP was identified in the aligned CDS but no resulting amino acid variation occurred in the corresponding position of alignment, this site was defined as an identical site within the ortholog. To further understand phylogenetic relationships among A_1_, A_2_ and A_t1_, we focused on specific recombination regions to infer gene trees according to a previous report^62^. We applied reported methods^63^ to further perform phylogenetic analysis among A_1_, A_2_ and A_t1_ or A_t2_. In brief, genome alignments were divided into 10-kb segments and we performed an approximately unbiased test. The site likelihoods for each possible topology were calculated by RAXML, then these likelihoods were input into Consel^64^.

### SNP identification

The sequenced reads of 14 A_1_ and 67 A_2_ cotton accessions were mapped to our assembled A_2_-genome in this study using BWA (0.7.10-r789)^65^. PCR duplications in the alignments were removed in Picard (v.1.94). SNPs and indels identified by the HaplotypeCaller module were then used to perform base-quality recalibration with the BaseRecalibrator and IndelRealigner modules in the GATK toolkit (v.3.8)^66^. The genomic variants in GVCF (genomic variant call format) for each accession as identified by the HaplotypeCaller module and the GVCF files were merged. Raw SNP calls were further filtered using GATK filter expressions (‘QUAL<30.0||QD<2.0||FS>60.0||MQ<40.0||SOR>4.0’ --clusterWindowSize 5 --clusterSize 2).

### Population genetics analysis

A subset of 9,555,165 SNPs (max-missing > 0.5, minor allele frequency > 0.05) in the 14 A_1_ and 67 A_2_ cotton accessions was screened to build a neighbor-joining tree in MEGA7 (ref. ^67^) with 1,000 bootstrap replicates using D_5_ as the outgroup. The cotton population structure analysis and a PCA were carried out using admixture^68^ with *K* values from 2 to 3 and EIGENSOFT software^69^, respectively. A pairwise fixation statistic (*F*_ST_) analysis as calculated in the PopGenome package^70^ was used to estimate the degree of variability in three groups (A_1_ accessions worldwide, A_2_ accessions from the India and Pakistan group, and A_2_ accessions from the China group). To validate the relationships of the A_1_, A_2_ and A_t1_, we used 30 released (AD)_1_ accessions^32,71^, 21 released A_2_ accessions and 14 released A_1_ accessions to construct a population phylogenetic tree with D_5_ as the outgroup (Extended Data Fig. 7). The identification of ancestral alleles was as described^18^.

### Phylogenetic weighting

For genome-wide evaluation of three possible phylogenetic hypotheses, a method called Twisst^72,73^ was applied to analyze A_1_ and A_2_ accessions. In brief, the phasing and imputation of filtered SNPs (minAlleles 2, depth (DP) ≥ 5, genotype quality (GQ) ≥ 30) obtained from the 14 A_1_ and 67 A_2_ accessions and the outgroup D_5_ were performed using Beagle software with default parameters. Trees were constructed for each sliding window of 50 SNPs across 13 chromosomes using Phyml software, then tree weightings were computed using Twisst, with four defined taxa: D_5_, A_1_ and A_2_ from China, and from India/Pakistan.

### Demographic analysis

The G-PhoCS^74^ method was employed to infer the complete demographic history for A_1_, A_2_ and A_t1_ based on 2,468 selected neutral loci. Coalescence simulations were run under two models, M1 (no gene flow) and M2 (ancient gene flow). To further convert estimates of divergence time (*τ*) and population size (*θ*) from mutations per site to years (*T*) and effective numbers of individuals (*N*_e_), respectively, we assumed an A_1_A_2_–A_t1_ average genomic divergence time of *T*_div_ ≈ 1.0 Ma (0.6–1.8 Ma), which was calculated by the molecular tree based on single-copy genes, and an annual production. We further applied *fastsimcoal2* software^75^ to infer demographic history based on fourfold degenerate sites selected from SNP datasets (minor allele frequencies > 0.05) from 30 released (AD)_1_ accessions, 21 released A_2_ accessions and 14 released A_1_ accessions.

### SVs among three cotton genomes

SVs were identified using NGMLR (v.0.2.4)^76^ and PbSV (v.0.1.0). First, we mapped the PacBio subreads of A_1_ and A_2_ to the genome of (AD)_1_ using NGMLR with default parameters, and then PbSV was used to find large indels with length >50 bp using parameters: gapdistance = 1,000, min_readcount = 2, min_readfraction = 0.2, positionwiggle = 200, basepairidwiggle = 0.25, call_min_mapq = 10.

### RT–qPCR analysis and plant transformation

Total RNA (~2 μg) was extracted and was then reverse transcribed in a 20-μl reaction mixture with TransScript cDNA Synthesis SuperMix (TransGen Biotech). Then 1-μl sample aliquots were used as templates for RT–qPCR analysis. *UBQ7* was used as the internal control for RT–qPCR data analysis. The CDS sequences of the *KCS6* gene were PCR amplified from the complementary DNA of 10-DPA fiber tissue and cloned into the pCAMBIA2300 vector, forming *35S::KCS6* or *E6::KCS6* constructs. Then the construct was introduced into *Agrobacterium tumefaciens* strain LBA4404, and subsequently transferred into the Upland cotton *G. hirsutum* cv. Zhong24. All primers used in this study are presented in Supplementary Table 16.

### Statistical analyses

Student’s two-tailed *t*-tests were performed in GraphPad Prism software.

### Reporting Summary

Further information on research design is available in the Nature Research Reporting Summary linked to this article.

## Online content

Any methods, additional references, Nature Research reporting summaries, source data, extended data, supplementary information, acknowledgements, peer review information; details of author contributions and competing interests; and statements of data and code availability are available at 10.1038/s41588-020-0607-4.

## Supplementary information

Supplementary Information Supplementary Figs. 1–3 and Tables 1–5, 15 and 16

Reporting Summary

Supplementary Table Supplementary Tables 6–14

## Data Availability

The genome sequence data for A_1_ and A_2_ are deposited in NCBI (PRJNA506494). The (AD)_1_-genome sequence data are accessible through NCBI (PRJNA524970). The assemblies and annotation files of A_1_, A_2_ and (AD)_1_ are available at the CottonGen website (https://www.cottongen.org/). The re-sequence data for A_1_ and A_2_ accessions can be accessed with accession number PRJNA507537 in NCBI. Source data for Figs. 2 and 5 and Extended Data Fig. 5 are presented with the paper.

## Source data

Source Data Fig. 2 Statistical Source Data
Source Data Fig. 5 Statistical Source Data
Source Data Extended Data Fig. 5 Unprocessed gels for Extended Data Fig. 5

## References

[1] Wu Z (2017). Cotton functional genomics reveals global insight into genome evolution and fiber development. J. Genet. Genomics.

[2] Ma Z (2018). Resequencing a core collection of upland cotton identifies genomic variation and loci influencing fiber quality and yield. Nat. Genet..

[3] Senchina DS (2003). Rate variation among nuclear genes and the age of polyploidy in *Gossypium*. Mol. Biol. Evol..

[4] Webber JM (1936). Cytogenetic notes on cotton and cotton relatives. II. Science.

[5] Zahn LM (2012). Unraveling the origin of cotton. Science.

[6] Stephens SG (1944). Phenogenetic evidence for the amphidiploid origin of New World cottons. Nature.

[7] Hutchinson, J. B., Silow, R. A. & Stephens, S. G. (eds) *The Evolution of* Gossypium *and the Differentiation of the Cultivated Cottons* (Oxford Univ. Press, 1947).

[8] Gerstel D (1953). Chromosomal translocations in interspecific hybrids of the genus *Gossypium*. Evolution.

[9] Palmer SA (2012). Archaeogenomic evidence of punctuated genome evolution in *Gossypium*. Mol. Biol. Evol..

[10] Hu Y (2019). *Gossypium barbadense* and *Gossypium hirsutum* genomes provide insights into the origin and evolution of allotetraploid cotton. Nat. Genet..

[11] Wendel, J. F., Brubaker, C., Alvarez, I., Cronn, R. & Stewart, J. M. *Genetics and Genomics of Cotton* Vol. 3 (ed. Paterson, A. H.) Ch. 1 (Springer, 2009).

[12] Endrizzi JE, Turcotte EL, Kohel RJ (1985). Genetics, cytology, and evolution of *Gossypium*. Adv. Genet..

[13] Wendel JF (1989). New World tetraploid cottons contain Old World cytoplasm. Proc. Natl Acad. Sci. USA.

[14] Kulkarni, V. N., Khadi, B. M., Maralappanavar, M. S., Deshapande, L. A. & Narayanan, S. S. *Genetics and Genomics of Cotton* Vol. 3 (ed. Paterson, A. H.) Ch. 4 (Springer, 2009).

[15] Wang K (2012). The draft genome of a diploid cotton *Gossypium raimondii*. Nat. Genet..

[16] Paterson AH (2012). Repeated polyploidization of *Gossypium* genomes and the evolution of spinnable cotton fibres. Nature.

[17] Li F (2014). Genome sequence of the cultivated cotton *Gossypium arboreum*. Nat. Genet..

[18] Du X (2018). Resequencing of 243 diploid cotton accessions based on an updated A genome identifies the genetic basis of key agronomic traits. Nat. Genet..

[19] Li FG (2015). Genome sequence of cultivated Upland cotton (*Gossypium hirsutum* TM-1) provides insights into genome evolution. Nat. Biotechnol..

[20] Zhang TZ (2015). Sequencing of allotetraploid cotton (*Gossypium hirsutum* L. acc. TM-1) provides a resource for fiber improvement. Nat. Biotechnol..

[21] Wang M (2019). Reference genome sequences of two cultivated allotetraploid cottons, *Gossypium hirsutum* and *Gossypium barbadense*. Nat. Genet..

[22] Hutchinson J (1954). New evidence on the origin of the Old World cottons. Heredity.

[23] Renny-Byfield S (2016). Independent domestication of two Old World cotton species. Genome Biol. Evol..

[24] Wang S (2015). Sequence-based ultra-dense genetic and physical maps reveal structural variations of allopolyploid cotton genomes. Genome Biol..

[25] Menzel MY, Brown MS (1954). The significance of multivalent formation in three-species *Gossypium* hybrids. Genetics.

[26] Watt, G. *The Wild and Cultivated Cotton Plants of the World* (Longmans, Green and Co., 1907).

[27] Teh BT (2017). The draft genome of tropical fruit durian (*Durio zibethinus*). Nat. Genet..

[28] Argout X (2011). The genome of *Theobroma cacao*. Nat. Genet..

[29] Islam MS (2017). Comparative genomics of two jute species and insight into fibre biogenesis. Nat. Plants.

[30] Gao Y (2018). De novo genome assembly of the red silk cotton tree (*Bombax ceiba*). GigaScience.

[31] Sun S (2018). Extensive intraspecific gene order and gene structural variations between Mo17 and other maize genomes. Nat. Genet..

[32] Fang L (2017). Genomic analyses in cotton identify signatures of selection and loci associated with fiber quality and yield traits. Nat. Genet..

[33] Qin YM (2007). Saturated very-long-chain fatty acids promote cotton fiber and *Arabidopsis* cell elongation by activating ethylene biosynthesis. Plant Cell.

[34] Xiao GH, Wang K, Huang G, Zhu YX (2015). Genome-scale analysis of the cotton KCS gene family revealed a binary mode of action for gibberellin A regulated fiber growth. J. Integr. Plant Biol..

[35] Wendel, J. F. et al. *Polyploidy and Genome Evolution* (eds Soltis, P. S. & Soltis, D. E.) Ch. 10 (Springer, 2012).

[36] Ling HQ (2018). Genome sequence of the progenitor of wheat A subgenome *Triticum urartu*. Nature.

[37] Banks JA (2011). The *Selaginella* genome identifies genetic changes associated with the evolution of vascular plants. Science.

[38] Chin CS (2016). Phased diploid genome assembly with single molecule real-time sequencing. Nat. Methods.

[39] Chin CS (2013). Nonhybrid, finished microbial genome assemblies from long-read SMRT sequencing data. Nat. Methods.

[40] Walker BJ (2014). Pilon: an integrated tool for comprehensive microbial variant detection and genome assembly improvement. PLoS ONE.

[41] English AC (2012). Mind the gap: upgrading genomes with Pacific Biosciences RS long-read sequencing technology. PLoS ONE.

[42] Benson G (1999). Tandem repeats finder: a program to analyze DNA sequences. Nucleic Acids Res..

[43] Tarailo-Graovac M, Chen N (2009). Using RepeatMasker to identify repetitive elements in genomic sequences. Curr. Protoc. Bioinformatics.

[44] Kapitonov VV, Jurka J (2008). A universal classification of eukaryotic transposable elements implemented in Repbase. Nat. Rev. Genet..

[45] Xu Z, Wang H (2007). LTR_FINDER: an efficient tool for the prediction of full-length LTR retrotransposons. Nucleic Acids Res..

[46] Keilwagen J (2016). Using intron position conservation for homology-based gene prediction. Nucleic Acids Res..

[47] Kim D, Langmead B, Salzberg SL (2015). HISAT: a fast spliced aligner with low memory requirements. Nat. Methods.

[48] Pertea M (2015). StringTie enables improved reconstruction of a transcriptome from RNA-seq reads. Nat. Biotechnol..

[49] Korf I (2004). Gene finding in novel genomes. BMC Bioinformatic.

[50] Stanke M (2006). AUGUSTUS: ab initio prediction of alternative transcripts. Nucleic Acids Res..

[51] Burge C, Karlin S (1997). Prediction of complete gene structures in human genomic DNA. J. Mol. Biol..

[52] Majoros WH, Pertea M, Salzberg SL (2004). TigrScan and GlimmerHMM: two open source ab initio eukaryotic gene-finders. Bioinformatics.

[53] Haas BJ (2008). Automated eukaryotic gene structure annotation using EVidenceModeler and the program to assemble spliced alignments. Genome Biol..

[54] Bateman A (2015). UniProt: a hub for protein information. Nucleic Acids Res..

[55] Moriya Y, Itoh M, Okuda S, Yoshizawa AC, Kanehisa M (2007). KAAS: an automatic genome annotation and pathway reconstruction server. Nucleic Acids Res..

[56] Jones, P. et al. InterProScan 5: genome-scale protein function classification. *Bioinformatics* **30**, 1236–1240 (2014). 10.1093/bioinformatics/btu031 PMC3998142 24451626

[57] Ashburner, M. et al. Gene Ontology: tool for the unification of biology. *Nat. Genet*. **25**, 25–29 (2000). 10.1038/75556 PMC3037419 10802651

[58] Li L, Stoeckert CJ, Roos DS (2003). OrthoMCL: identification of ortholog groups for eukaryotic genomes. Genome Res..

[59] Katoh K, Standley DM (2013). MAFFT multiple sequence alignment software version 7: improvements in performance and usability. Mol. Biol. Evol..

[60] Stamatakis A (2014). RAxML version 8: a tool for phylogenetic analysis and post-analysis of large phylogenies. Bioinformatics.

[61] Yang Z (1997). PAML: a program package for phylogenetic analysis by maximum likelihood. Comput. Appl. Biosci..

[62] Pease JB, Hahn MW (2013). More accurate phylogenies inferred from low-recombination regions in the presence of incomplete lineage sorting. Evolution.

[63] Schumer M, Cui R, Powell DL, Rosenthal GG, Andolfatto P (2016). Ancient hybridization and genomic stabilization in a swordtail fish. Mol. Ecol..

[64] Shimodaira H, Hasegawa M (2001). CONSEL: for assessing the confidence of phylogenetic tree selection. Bioinformatics.

[65] Li H, Durbin R (2009). Fast and accurate short read alignment with Burrows–Wheeler transform. Bioinformatics.

[66] McKenna A (2010). The genome analysis toolkit: a MapReduce framework for analyzing next-generation DNA sequencing data. Genome Res..

[67] Kumar S, Stecher G, Tamura K (2016). MEGA7: molecular evolutionary genetics analysis version 7.0 for bigger datasets. Mol. Biol. Evol..

[68] Alexander DH, Novembre J, Lange K (2009). Fast model-based estimation of ancestry in unrelated individuals. Genome Res..

[69] Price AL (2006). Principal components analysis corrects for stratification in genome-wide association studies. Nat. Genet..

[70] Pfeifer B, Wittelsbürger U, Ramos-Onsins SE, Lercher MJ (2014). PopGenome: an efficient Swiss army knife for population genomic analyses in R. Mol. Biol. Evol..

[71] Wang M (2017). Asymmetric subgenome selection and *cis*-regulatory divergence during cotton domestication. Nat. Genet..

[72] Martin SH, Van Belleghem SM (2017). Exploring evolutionary relationships across the genome using topology weighting. Genetics.

[73] Van Belleghem SM (2017). Complex modular architecture around a simple toolkit of wing pattern genes. Nat. Ecol. Evol..

[74] Gronau I, Hubisz MJ, Gulko B, Danko CG, Siepel A (2011). Bayesian inference of ancient human demography from individual genome sequences. Nat. Genet..

[75] Excoffier L, Dupanloup I, Huerta-Sanchez E, Sousa VC, Foll M (2013). Robust demographic inference from genomic and SNP data. PLoS Genet..

[76] Fritz JS (2018). Accurate detection of complex structural variations using single-molecule sequencing. Nat. Methods.

