# Supplementary material for: Genome sequence of Gossypium herbaceum and genome updates of Gossypium arboreum and Gossypium hirsutum provide insights into cotton A-genome evolution
Source: Nat Genet. 2020 Apr 13;52(5):516–24. doi: 10.1038/s41588-020-0607-4 (PMC7203013; doi:10.1038/s41588-020-0607-4)
Supplement: Supplementary file 1 — Supplementary Figs. 1–3 and Tables 1–5, 15 and 16 [file 41588_2020_607_MOESM1_ESM.pdf]

nature genetics

SUPPLEMENTARY INFORMATION

https://doi.org/10.1038/s41588-020-0607-4

In the format provided by the authors and unedited.

OPEN

Genome sequence of *Gossypium herbaceum* and genome updates of *Gossypium arboreum* and *Gossypium hirsutum* provide insights into cotton A-genome evolution

Gai Huang[1,2,7], Zhiguo Wu[3,7], Richard G. Percy[4], Mingzhou Bai[5], Yang Li[3], James E. Frelichowski[4], Jiang Hu[6], Kun Wang[3], John Z. Yu[4]✉ and Yuxian Zhu[1]✉

[1]Institute for Advanced Studies, Wuhan University, Wuhan, China. [2]State Key Laboratory of Protein and Plant Gene Research, School of Life Sciences, Peking University, Beijing, China. [3]College of Life Sciences, Wuhan University, Wuhan, China. [4]Crop Germplasm Research Unit, Southern Plains Agricultural Research Center, United States Department of Agriculture-Agricultural Research Service (USDA-ARS), College Station, TX, USA. [5]BGI Genomics, BGI-Shenzhen, Shenzhen, China. [6]Nextomics Biosciences Institute, Wuhan, China. [7]These authors contributed equally: Gai Huang, Zhiguo Wu.
✉e-mail: john.yu@usda.gov; zhuyx@whu.edu.cn

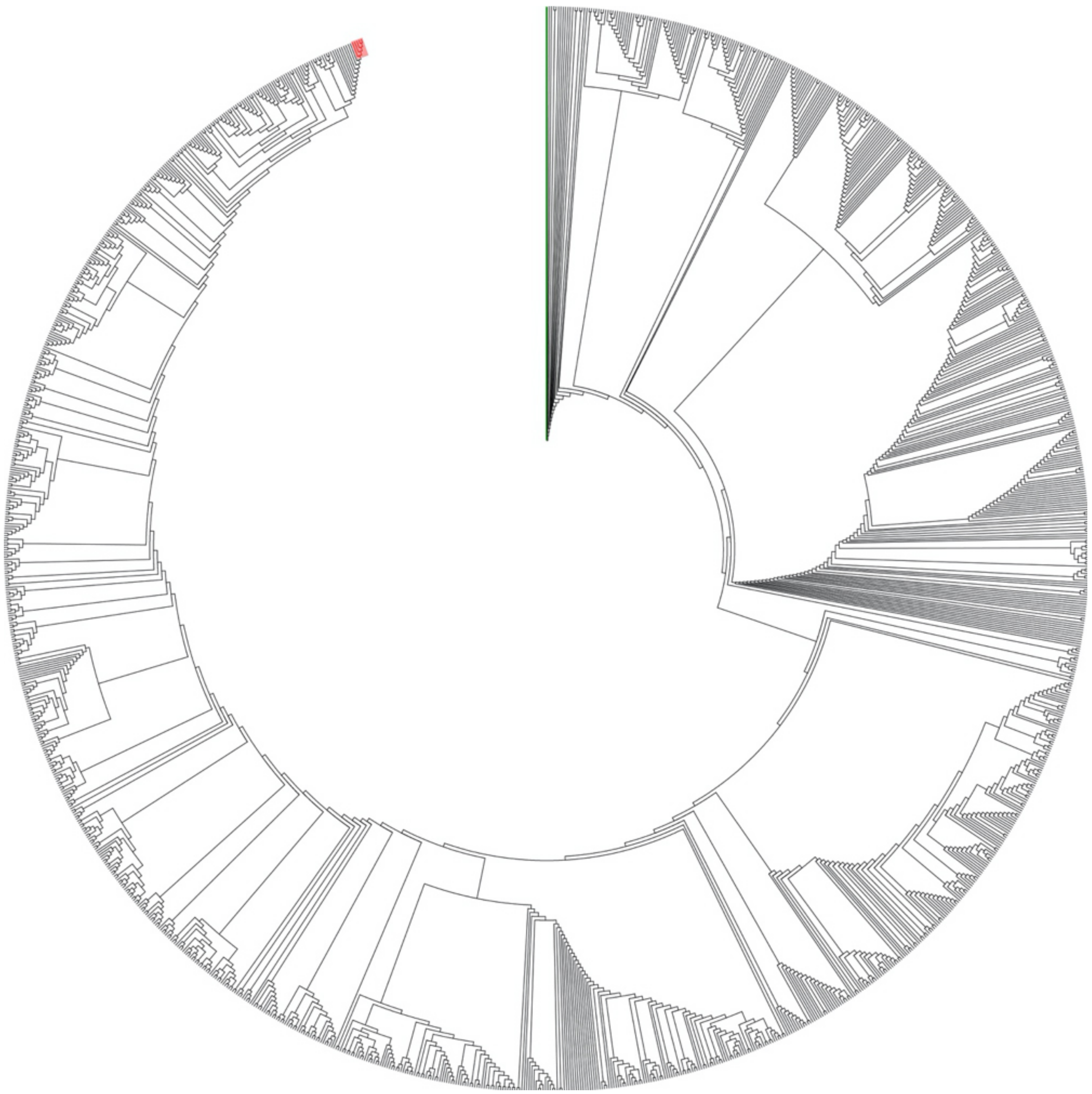

Supplementary Figure 1 Phylogenetic analysis of 1,397 Gypsy sequences with amino acids > 1000. We used one Copia sequence (green color) as the outgroup in this analysis. One of the five identical Gypsy sequences with 4,725 nt in length (red color) evolved most recently was chosen as the query sequence to scan different cotton genomes to evaluate TE hits by BLASTN (E-value ≤ 1e-5).

a b

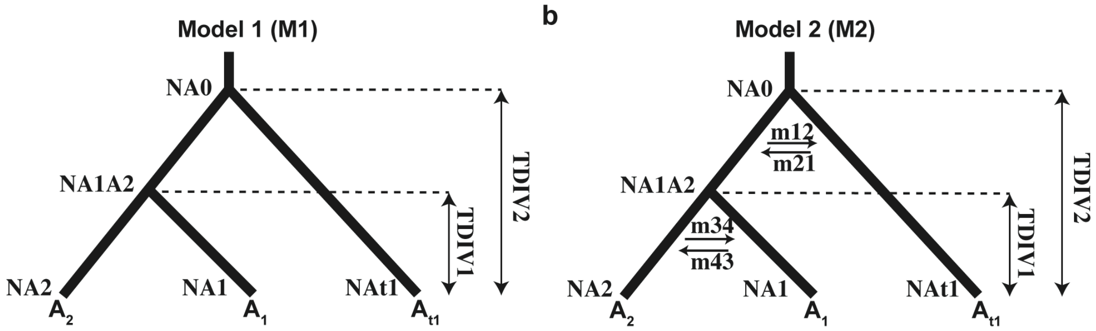

c

Model	Population size					Divergent time		Migration fraction				Max. EstLhood
	NA0	NA1A2	NA1	NA2	NAt1	TDIV1	TDIV2	m12	m21	m34	m43	
M1	235,733,419	68,712,614	480,606	1,159,292	11,738	1,016,499	1,444,136	–	–	–	–	-6.611
M2	245,272,923	108,058,594	576,408	1,344,231	60,963	1,240,448	2,009,279	7.98E-07	5.28E-10	2.66E-09	2.09E-09	-6.622

d e

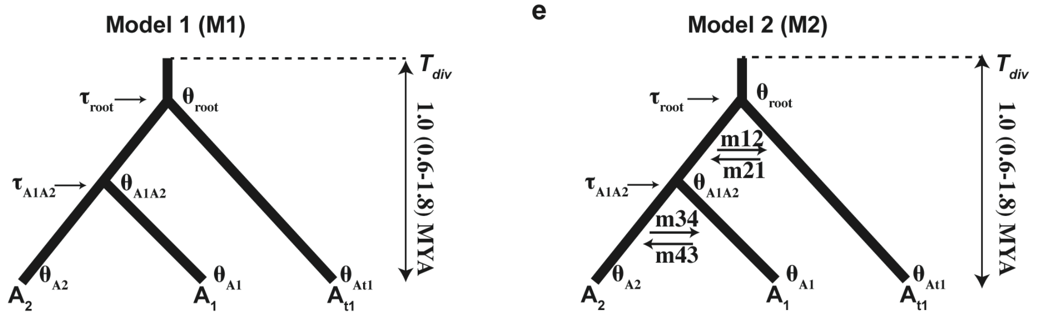

f

Model		Population size					Divergent time		Migration rate				Full-ld-ln
		θ_{root}	θ_{A1A2}	θ_{A1}	θ_{A2}	θ_{At1}	τ_{root}	τ_{A1A2}	m12	m21	m34	m43	
M1	Raw values	1279.70	368.13	297.28	370.63	1.15	2558.68	521.26	–	–	–	–	-8385295.87
	Converted values	125035 (75021-225064)	35969 (21581-64744)	29046 (17427-52282)	36213 (21728-65184)	112 (67-202)	1.0 (0.6-1.8)	0.2 (0.1-0.4)					
M2	Raw values	979.76	314.93	292.40	368.44	1.71	2925.88	566.24	1.29E-4	1.89E-4	4.90E-4	3.06E-4	-8384352.40
	Converted values	83715 (50229-150688)	26909 (16145-48436)	24984 (14990-44970)	31482 (18889-56667)	146 (88-263)	1.0 (0.6-1.8)	0.2 (0.1-0.3)					

Supplementary Figure 2 Demographic inference results from ***fastsimcoal2*** **and G-PhoCS. a-c**, The assumed two models (**a-b)** and corresponding results (**c**) imputed from *fastsimcoal2* software. The absolute population sizes (NA0, NA1A2, NA1, NA2, NAt1), the divergence time (TDIV1 and TDIV2), and migration fraction (m12, m21, m34, m43) were represented in the diagram. Max. EstLhood: log-likelihood of the best estimate. **d-f**, The assumed two models (**d-e)** and corresponding results (**f**) imputed from G-PhoCS software. The free parameters in our models include divergence times (τ_{root} and τ_{A1A2}), effective population sizes (θ_{root}, θ_{A1A2}, θ_{A1}, θ_{A2}, θ_{At1}), and migration rate (m12, m21, m34, m43) were shown in the diagram. Full-ld-ln, log likelihood across all loci. We assumed a A_1A_2-A_{t1} average genomic divergence time of T_{div} = 1.0 MYA (0.6-1.8 MYA). The values in the upper and bottom for each model indicate the raw estimated values and converted calibrated values in million years ago or number of individuals, respectively.

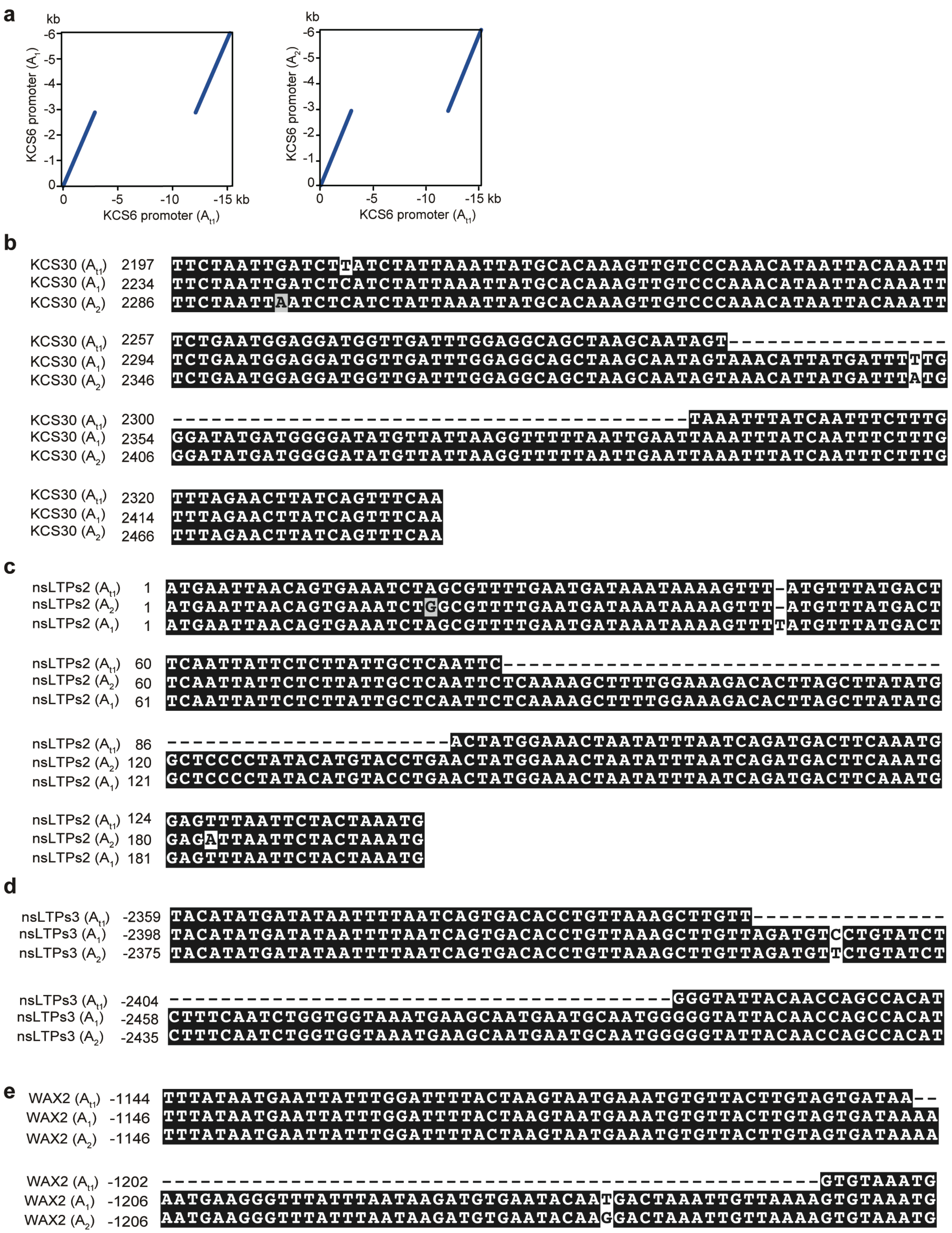

Supplementary Figure 3 Structural variations analysis of genes involved in fatty acid biosynthesis. a, Dot plots showing the pairwise comparison in the promoter of *KCS6* gene between A$_{t1}$ and A$_1$ (left panel) or A$_2$ (right panel). **b, c,** Sequence alignments of the downstream regions of *KCS30* (**b**) and *nsLTPs2* (**c**) genes in A$_{t1}$, A$_1$ and A$_2$. **d, e,** Sequence alignments of the promoters of *nsLTPs3* (**d**) and *WAX2* (**e**) genes in A$_{t1}$, A$_1$ and A$_2$.

Supplementary Table 1 Sequence statistics of A_1, A_2 and $(AD)_1$.

Species	Platform	Number of reads	Total bases (Gb)	Genome coverage
A_1-genome	Illumina paired-end reads	343,444,702	52	32
	PacBio long reads	23,281,921	225	138
	Hi-C data	665,246,160	256	-
A_2-genome	Illumina paired-end reads	826,656,862[a]	95	54
	PacBio long reads	14,021,348[b] + 19,909,768	310	177
	Hi-C data	1,093,220,857	219	-
$(AD)_1$-genome	Illumina paired-end reads	233,514,500	70	28.2
	PacBio long reads	21,620,284	205	81.6
	Hi-C data	628,486,772	196	-

[a]Previously released Illumina paired-end reads[17]; [b]Previously published PacBio long reads[18].

Supplementary Table 2 The statistics of annotated genes based on different databases.

Database	A_1-genome		A_2-genome		$(AD)_1$-genome	
	Genes[a]	%[b]	Genes[a]	%[b]	Genes[a]	%[b]
KEGG	13,088	29.78	12,754	29.47	54,749	73.56
InterProScan	32,202	73.27	32,626	75.39	63,583	85.43
GO	22,622	51.47	22,662	52.36	47,138	63.33
Swiss-Prot	31,489	71.64	31,572	72.95	56,570	76.01
TrEMBL	41,601	94.65	41,595	96.11	71,353	95.87
Annotated Genes	42,088	95.76	41,963	96.96	71,880	96.58
Unannotated Genes	1,864	4.24	1,315	3.04	2,470	3.32
**Total Genes**	**43,952**	**-**	**43,278**	**-**	**74,350**	**-**

[a]Number of annotated genes; [b]Percentage of annotated genes.

Supplementary Table 3 The content of major transposable element (TE) subfamilies in A_1, A_2 and $(AD)_1$ genome.

Type	A_1-genome			A_2-genome			$(AD)_1$-genome		
	TEs	Length (bp)	%	TEs	Length (bp)	%	TEs	Length (bp)	%
**LTR**	561,747	1,129,188,947	72.57	609,628	1,200,147,210	73.62	1,080,168	1,425,793,804	62.26
DIRS	33	16,955	0	39	17,637	0	49	165,866	0.01
non	30,034	358,817,234	23.06	34,708	403,844,113	24.77	223,771	181,560,601	7.93
ERVL	56	25,042	0	44	29,929	0	48	36,500	0
ERV-Foamy	2	1,317	0	13	5,478	0	0	0	0
Foamy	57	33,991	0	67	35,571	0	0	0	0
Copia	63,843	51,801,339	3.33	71,167	50,370,643	3.09	224,839	180,197,390	7.87
Caulimovirus	2,005	1,691,541	0.11	2,555	1,567,477	0.09	2,351	3,474,185	0.15
ERVK	16	9,088	0	30	13,815	0	135	131,228	0.01
Gypsy	464,988	765,924,850	49.22	500,182	807,477,548	49.53	628,297	1,245,839,249	54.4
ERV4	9	12,107	0	6	2,679	0	0	0	0
Lenti	3	2,064	0	1	1,011	0	0	0	0
ERV1	501	262,696	0.02	564	296,579	0.02	170	297,738	0.01
Cassandra	2	175	0	27	3,264	0	28	205,680	0.01
Gypsy-Cigr	158	79,007	0.01	176	86,965	0.01	2	1,437	0
Pao	40	13,458	0	47	14312	0	478	275,834	0.01
**Satellite**	414	89,737	0.01	427	90,958	0.01	260	435,007	0.02
**SINE**	749	165,122	0.01	44	4,444	0	652	515,466	0.02
**DNA**	79,715	32,595,860	2.09	95,753	34,310,457	2.1	52,932	38,902,101	1.70
**Simple repeat**	144,505	27,790,077	1.79	144,150	24,746,670	1.52	15,382	14,483,315	0.63
**Unknown**	207,846	46,690,876	3	192,330	41,410,307	2.54	19,328	12,037,493	0.53
**Retroposon**	5	303	0	7	547	0	0	0	0
**ARTEFACT**	5	1,046	0	3	129	0	0	0	0
**Other**	5	759	0	6	813	0	74	64,435	0

**RC**	2,853	741,440	0.05	4,706	858,355	0.05	5,887	3,140,472	0.14
**LINE**	21,205	15,540,701	1	22,357	15,246,706	0.94	20,768	15,334,318	0.67
**Low complexity**	46	5,648	0	48	7,460	0	0	0	0
**Total TEs**	**-**	**1,240,337,705**	**79.71**	**-**	**1,305,128,430**	**80.06**	**-**	**1,467,548,098**	**64.09**

Supplementary Table 4 Summary of sequencing and resequencing data for A_1, A_2 and $(AD)_1$ accessions.

Sample	Germplasm name	Origin	Total reads	Depth	Source
Gar01	Malvi 9	Madhya Pradesh, India	88,213,648	7	this study
Gar02	Koresu	Iran thru USSR	112,818,744	9	this study
Gar03	LINE O-824 II-I	Uzbekistan	130,802,220	10	this study
Gar04	var. soudanense	West Iran thru CSSR	122,585,774	10	this study
Gar05	AnHuiShuChengZhongMian	Anhui, China	64,511,506	5	SRR4457103
Gar06	ZheJiangJinHuaZhongMian	Zhejiang, China	78,603,284	5	SRR4457099
Gar07	XinGanZhongMian	Jiangxi, China	79,959,328	5	SRR4457092
Gar08	XiaoBaiHuaZhongMian	Hebei, China	76,189,048	5	SRR4457089
Gar09	ZiYunXiaoHua	Guizhou, China	71,555,798	5	SRR4457087
Gar10	GeBeiMian	Guangdong, China	66,792,870	5	SRR4457084
Gar11	ChangShuXiaoBaiZi	Jiangsu, China	65,121,918	5	SRR4457052
Gar12	SanJiangBaJiangZhongMian	Guangxi, China	66,442,248	5	SRR4457048
Gar13	ZhongMian-6	Liaoning, China	92,712,914	6	SRR4457033
Gar14	119S	Pakistan	60,284,702	4	SRR4456990
Gar15	XiaoGanTieZiXiaoYiHua	Hubei, China	66,966,898	5	SRR4456978
Gar16	IndiaN.V50-70	India	76,455,914	5	SRR4456964
Gar17	MoJiangNaHaZhongMian	Yunnan, China	67,785,654	5	SRR4456963
Gar18	SuiXianZiHuaMian	Henan, China	74,851,628	5	SRR4456924
Gar19	SiChuanFuShunZhongMian	Sichuan, China	65,117,104	5	SRR4456916
Gar20	ChangZi-1	Hunan, China	77,869,404	5	SRR4456908
Gar21	FuJianShenShanXiangZiHuaZhongMian	Fujian, China	56,874,352	4	SRR4456904
Gar22	SongJiangBaiZi	Shanghai, China	64,598,176	5	SRR4456900
Gar23	NongLin-4	Japan	65,714,858	4	SRR4456897
Gar24	BaiMiLaHuangHuaHeiZi	Japan	81,188,176	6	SRR4456894
Gar25	DuiHua	Shandong, China	70,315,626	5	SRR4456893
Gar26	GanSuWuDuZhongMian	Gansu, China	47,750,614	3	SRR4456887
Gar27	Dharwar cotton	India	74,568,232	5	SRR4456884
Gar28	ShanXiLinFenDongMaCeZhongMian	Shanxi, China	81,787,060	6	SRR4456872
Gar29	shixiya	China	100,463,068	8	SRR1216982
Gar30	DC-92	Lyallpur, Pakistan	126,630,572	10	this study
Gar31	Indicum 38	Maharashtra, India	111,540,600	9	this study
Gar32	Dhulia 215	Maharashtra, India	122,112,020	10	this study
Gar33	Burma Silk	Maharashtra, India	121,147,820	10	this study
Gar34	Kokati Khaki	Maharashtra, India	113,516,452	9	this study
Gar35	Indicum 10	Maharashtra, India	121,257,814	10	this study
Gar36	var. burmanicum	Burma	124,840,436	10	this study
Gar37	Jarilla	New Delhi, India	126,209,070	10	this study

Gar38	White Flower	Pakistan thru USSR	116,253,318	9	this study
Gar39	var. cernuum	-	102,780,502	8	this study
Gar40	var. indicum	India	107,218,130	8	this study
Gar41	Verum	Madhya Pradesh, India	130,489,450	10	this study
Gar42	Nanking	Tashkent, Uzbekistan	112,042,660	9	this study
Gar43	Kapas	Rajastan, India	100,522,446	8	this study
Gar44	Desi Rui	India	109,410,374	9	this study
Gar45	An. B.B. 1000P7	Pakistan	109,244,490	9	this study
Gar46	Kapas	Rajastan, India	123,049,598	10	this study
Gar47	var. roseum	Pakistan thru USSR	124,950,442	10	this study
Gar48	var. bengalense	-	121991878	10	this study
Gar49	var. bengalense	-	123,596,110	10	this study
Gar50	var. bengalense	-	135,138,492	11	this study
Gar51	var. carnuum	-	92,532,676	7	this study
Gar52	var. bengalense	-	96,632,470	8	this study
Gar53	var. carnuum	-	111,467,542	9	this study
Gar54	var. indicum	India	101,623,554	8	this study
Gar55	var. bengalense	-	95,449,088	8	this study
Gar56	var. bengalense	-	120,558,800	10	this study
Gar57	var. indicum	India	108,518,874	8	this study
Gar58	var. indicum	-	112,508,838	8	this study
Gar59	var. bengalense	-	114,229,450	9	this study
Gar60	var. cernuum	India	118,358,560	9	this study
Gar61	Red Flower	Pakistan thru USSR	141,024,176	11	this study
Gar62	var. burmanicum	-	119,348,234	9	this study
Gar63	var. bengalense	-	110,875,780	9	this study
Gar64	var. bengalense	India	110,734,970	9	this study
Gar65	var. soudanense	West Iran thru India	96,000,786	8	this study
Gar66	var. indicum	India	122,122,052	9	this study
Gar67	Nha92	Vietnam	87,546,296	6	SRR4456883
Ghe01	var. africanum (Mutema, A1-0076)	Kodoma, ZIMBABWE	82,420,578	7	this study
Ghe02	Russian No. 2531	Russian	82,505,072	7	this study
Ghe03	Local cotton	-	73,076,198	5	SRR4456980
Ghe04	var. africanum	South Africa	63,337,200	4	SRR4456979
Ghe05	Gusa	Former Soviet Union	83,284,082	6	SRR4456962
Ghe06	L-02066-1-3	Former Soviet Union	76,726,310	5	SRR4456961
Ghe07	CaoMian	Gansu, China	79,594,852	6	SRR4456959
Ghe08	ZiHuaCaoMian	Gansu, China	72,293,092	5	SRR4456957
Ghe09	HongKeCaoMian	Xinjiang, China	83,581,244	6	SRR4456956
Ghe10	LinZeCaoMian	Gansu, China	67,351,536	5	SRR4456955
Ghe11	HongXingCaoMian	China	62,000,450	4	SRR4456953
Ghe12	YuMenCaoMian	Gansu, China	70,335,344	5	SRR4456952
Ghe13	GaoTaiCaoMian	Gansu, China	73,715,176	5	SRR4456928
Ghe14	JinTaCaoMian	Gansu, China	86,152,064	6	SRR4456927
Ghi01	Apr-86	Beijing, China	51,443,904	6.2	SRR4013615

Ghi02	Dunmian1	Gansu, China	52,468,101	6.3	SRR4013885
Ghi03	Jinmian 2	Liaoning, China	52,054,631	6.2	SRR4014023
Ghi04	Lumian 5	Shandong, China	51,542,449	6.2	SRR4018461
Ghi05	Qinyuan 4	Shanxi, China	53,343,391	6.4	SRR4018537
Ghi06	Stoneville 2B	US	52,329,236	6.3	SRR4018547
Ghi07	Wanmian 17	Anhui, China	65,153,217	7.8	SRR4018553
Ghi08	Xumian18	Jiangsu, China	55,083,985	6.6	SRR4018580
Ghi09	Yanmian1	Sichuan, China	55,829,955	6.7	SRR4018583
Ghi10	Yun87-509	Shanxi, China	53,426,915	6.4	SRR4018594
Ghi11	Zhemian11	Zhejiang, China	57,086,290	6.9	SRR4018595
Ghi12	Xinluzao 22	the North of Xinjiang, China	63,882,582	7.7	SRR4018953
Ghi13	Emian 22	Hubei, China	57,639,793	6.9	SRR4018960
Ghi14	Xinluzhong 12	the South of Xinjiang, China	64,212,136	7.7	SRR4018964
Ghi15	CIR35	Yellow River, China	62,063,504	5.0	SRR1580613
Ghi16	Brazi011	Brazil	106,212,428	8.5	SRR1580636
Ghi17	MA-6	China	84,265,888	6.7	SRR1580644
Ghi18	CIR10	Yellow River, China	98,607,076	7.9	SRR1580610
Ghi19	Dixie Triumph	US	75,361,418	6.0	SRR1580591
Ghi20	Mebane	US	53,326,084	4.3	SRR1580599
Ghi21	Dixie King	US	101,029,278	8.1	SRR1580590
Ghi22	Tashkent 1	Xinjiang, China	71,757,441	5.7	SRR1580646
Ghi23	Punctatum 5	Mexico	63,033,417	5.0	SRR1580654
Ghi24	Morrilli 2	Mexico	44,251,533	3.5	SRR1580662
Ghi25	Yucatanense 1	Mexico	67,586,264	5.4	SRR1580665
Ghi26	Richmondi 3	Mexico	56,244,747	4.5	SRR1580674
Ghi27	Marie-galante 7	Mexico	60,433,621	4.8	SRR1580676
Ghi28	Latifolium 13	Mexico	71,682,491	5.7	SRR1580688
Ghi29	Palmeri 5	Mexico	110,242,849	8.8	SRR1580698
Ghi30	Ganmian12	Jingxi, China	63,665,192	7.6	SRR4006719

*Gar, *G. arboreum*; Ghe, *G. herbaceum*; Ghi, *G. hirsutum*; SRR, NCBI Sequence Read Archive accession number.

Supplementary Table 5 Statistics of calling variations in *G. herbaceum* and *G. arboreum* accessions in this study.

Region	Category	SNPs		INDELs	
		Numbers	Affected genes	Numbers	Affected genes
Intergenic	--	9,402,612	--	1,363,109	--
Up/Downstream (5 kb)	--	1,744,165	43,121	180,681	42,691
Intronic	--	316,718	25,182	158,777	21,021
Splicing	--	841	782	453	363
Exonic	Stop gain	2,830	2,451	553	461
	Stop loss	662	651	72	68
	Synonymous	75,575	28,273	--	--
	Non-synonymous	109,001	31,818	--	--
	Frameshift	--	--	8,730	5,696
	non-frameshift	--	--	4,533	3,153
**Total**	--	**11,652,404**	**43,139**	**1,716,908**	**42,733**

Supplementary Table 15 RNA-seq data used in this study.

Species	Tissue	No. of clean reads	Mapping rate	Source	Application
A_1	Root	29,019,936	93.42	SRR10609507	RNA-seq assisted gene predictions
	Stem	26,270,643	93.91	SRR10609506	
	Leaf	27,853,361	94.68	SRR10609505	
	Flower	31,967,950	92.62	SRR10609504	
	10 DPA ovule with fibers	37,098,220	92.55	SRR10609503	
	15 DPA ovule with fibers	30,125,170	92.61	SRR10609502	
	20 DPA ovule with fibers	33,791,494	93.27	SRR10609501	
	15 DPA fibers (replicate 1)	67,138,936	83.58	SRR10609500	Differential expression analysis
	15 DPA fibers (replicate 2)	67,817,539	83.72	SRR10609499	
	15 DPA fibers (replicate 3)	67,497,808	83.64	SRR10609498	
A_2	Root	40,312,381	89.34	SRR10609509	RNA-seq assisted gene predictions
	Stem	46,068,529	92.46	SRR10609508	
	Leaf	48,058,994	95.48	SRR10609497	
	Flower	38,784,777	92.63	SRR10609488	
	10 DPA ovule with fibers	35,556,430	91.42	SRR10609487	
	15 DPA ovule with fibers	33,578,748	89.95	SRR10609486	
	20 DPA ovule with fibers	39, 345,800	92.94	SRR10609485	
	15 DPA fibers (replicate 1)	23,121,783	96.97	SRR10609484	Differential expression analysis
	15 DPA fibers (replicate 2)	22,171,046	97.09	SRR10609483	
	15 DPA fibers (replicate 3)	21,291,992	97.23	SRR10609482	
$(AD)_1$	Root	20,930,856	88.21	SRR1695173*	RNA-seq assisted gene predictions
	Stem	10,871,958	91.45	SRR1695174*	
	Leaf	69,550,066	94.64	SRR10609496	
	Flower	60,105,180	89.92	SRR10609495	
	10 DPA ovule with fibers	59,210,571	94.59	SRR10609494	
	15 DPA ovule with fibers	28,880,150	94.66	SRR10609493	
	20 DPA ovule with fibers	56,231,616	96.34	SRR10609492	
	15 DPA fibers (replicate 1)	18,421,143	92.57	SRR10609491	Differential expression analysis
	15 DPA fibers (replicate 2)	19,800,871	92.88	SRR10609490	
	15 DPA fibers (replicate 3)	19,909,307	92.07	SRR10609489	

*Previously released data[20].

Supplementary Table 16 Primers used in this study.

Primer name	Gene id	Sequence (5'–3')	Application
F1	-	GGGGAATTTAACGGCTTGCA	To validate the borders of two large inversions in chromosomes 10 and 12 of A genomes
R1	-	GGCCTAACACACCTGGTTTG	
F1'	-	CGGGCCCATAAGAAACAAGG	
R1'	-	TGTTCCGCCTTCACTTGTTG	
F2	-	CGAATTGTCTAACGCTGGGG	
R2	-	TCTCTCCATGTTACTGCCGA	
F2'	-	TCAATCTGTCAACAAGTGGGT	
R2'	-	CGCTACCGCTTTGATCCATC	
F3	-	GGCCCGATCAACTAATGCAG	
R3	-	ATGGCTATGCTGTGAGTGGT	
F3'	-	GAGCCAATTCCCCTAGCCAT	
R3'	-	CTCGAGTCCTCTACCGCAC	
F4	-	AGCAGGCGACACTAGAGTTT	
R4	-	AAGCCTCCGTACACAGAGTT	
F4’	-	TGCTACACATACACATCCAA	
R4’	-	TTCATCAAGGTTCAGTTCCA	
UBQ7-F	Ghi_A11G05811	GGCATTCCACCTGACCAACAA	qRT-PCR analysis
UBQ7-R		CCGCATTAGGGCACTCTTTTC	
KCS6-F	Ghi_A05G01456	AGGTGTTATCAAGGGCAGGC	
KCS6-R		GCACTACACCCCATTCCAGT	
KCS30-F	Ghi_A10G13266	TGCGTGCGTGAAAGTTGTGTTG	
KCS30-R		CCATCTCGGCATGTGCATCGT	
nsLTPs2-F	Ghi_A05G20641	CTGATAGGGAAGCCTGGAAGTT	
nsLTPs2-R		TGCAGCGTTTTGGAATAGTGA	
nsLTPs3-F	Ghi_A10G09251	GGTGGTGTTGTGCATGGTAG	
nsLTPs3-F		CGGCGGAGTTGAGAGATTTG	
WAX2-F	Ghi_A05G19706	CCCTTCTCGCAATTCCCACCAA	
WAX2-R		TCCTCGGCAACCAGTTCTCACA	
WRKY12-F	Ghi_A02G05486	GCGTAGCTCTTCGGATCCTC	
WRKY12-R		TGCTTCTAGTACTTCCGGCG	
HD-Zip2-F	Ghi_A08G06296	TGAGCAGCACCAACTTAGGA	
HD-Zip2-R		GGAGGAATAGGACTACCAGAGG	
MYB6-F	Ghi_A10G09551	AACAGCGGTGATGGTATTGC	
MYB6-R		AGAACATTGGTGAATCCGATGG	
KCS6-CDS-F	Ghi_A05G01456	ATGGAGTCGTTCTCTTTTTTGTTG	Amplification of full-length CDS sequences by PCR
KCS6-CDS-R		TTAATCTCCGGGATTGAAGGT	
