# Supplementary material for: Genome sequence of Gossypium herbaceum and genome updates of Gossypium arboreum and Gossypium hirsutum provide insights into cotton A-genome evolution
Source: Nat Genet. 2020 Apr 13;52(5):516–24. doi: 10.1038/s41588-020-0607-4 (PMC7203013; doi:10.1038/s41588-020-0607-4)
Supplement: Supplementary file 2 — Reporting Summary [file 41588_2020_607_MOESM2_ESM.pdf]

nature research

Corresponding author(s): Yuxian Zhu

Last updated by author(s): Mar 1, 2020

Reporting Summary

Nature Research wishes to improve the reproducibility of the work that we publish. This form provides structure for consistency and transparency in reporting. For further information on Nature Research policies, see Authors & Referees and the Editorial Policy Checklist.

Statistics

For all statistical analyses, confirm that the following items are present in the figure legend, table legend, main text, or Methods section.

n/a	Confirmed	
☐	☒	The exact sample size (*n*) for each experimental group/condition, given as a discrete number and unit of measurement
☐	☒	A statement on whether measurements were taken from distinct samples or whether the same sample was measured repeatedly
☐	☒	The statistical test(s) used AND whether they are one- or two-sided<br>*Only common tests should be described solely by name; describe more complex techniques in the Methods section.*
☒	☐	A description of all covariates tested
☒	☐	A description of any assumptions or corrections, such as tests of normality and adjustment for multiple comparisons
☐	☒	A full description of the statistical parameters including central tendency (e.g. means) or other basic estimates (e.g. regression coefficient) AND variation (e.g. standard deviation) or associated estimates of uncertainty (e.g. confidence intervals)
☐	☒	For null hypothesis testing, the test statistic (e.g. *F*, *t*, *r*) with confidence intervals, effect sizes, degrees of freedom and *P* value noted<br>*Give P values as exact values whenever suitable.*
☒	☐	For Bayesian analysis, information on the choice of priors and Markov chain Monte Carlo settings
☒	☐	For hierarchical and complex designs, identification of the appropriate level for tests and full reporting of outcomes
☒	☐	Estimates of effect sizes (e.g. Cohen's *d*, Pearson's *r*), indicating how they were calculated

Our web collection on statistics for biologists contains articles on many of the points above.

Software and code

Policy information about availability of computer code

Data collection	PacBio reads were collected from single-molecule real-time (SMRT) cells on PacBio RSII and Sequel instruments; Hi-C data and pair-end reads were collected from the Illumina HiSeq platform.
Data analysis	All softwares and corresponding versions have been well described in the method.

For manuscripts utilizing custom algorithms or software that are central to the research but not yet described in published literature, software must be made available to editors/reviewers. We strongly encourage code deposition in a community repository (e.g. GitHub). See the Nature Research guidelines for submitting code & software for further information.

Data

Policy information about availability of data

All manuscripts must include a data availability statement. This statement should provide the following information, where applicable:

- Accession codes, unique identifiers, or web links for publicly available datasets
- A list of figures that have associated raw data
- A description of any restrictions on data availability

All software and web links used in this study were described in the method section. The genome sequence data for A1 and A2 are deposited in NCBI (PRJNA506494). The (AD)1-genome sequence data are accessible through NCBI (PRJNA524970). The re-sequence data for A1 and A2 accessions can be accessed with accession number PRJNA507537 in NCBI. Our genome assemblies and annotation files of A1, A2 and (AD)1 are available at the CottonGen website (https://www.cottongen.org/).

Field-specific reporting

Please select the one below that is the best fit for your research. If you are not sure, read the appropriate sections before making your selection.

☒ Life sciences ☐ Behavioural & social sciences ☐ Ecological, evolutionary & environmental sciences

For a reference copy of the document with all sections, see nature.com/documents/nr-reporting-summary-flat.pdf

Life sciences study design

All studies must disclose on these points even when the disclosure is negative.

Sample size	For population analysis, we collected 14 A1 and 67 A2 representative cotton accessions from India, Pakistan, China, and other countries; For the growth rate measurement of cotton fibers in different cotton species, 30 seeds for each species at the given time point were selected for measurement.
Data exclusions	No data were excluded.
Replication	For qRT-PCR analysis, three independent experiments were performed; For RNA-seq of elongating fibers at 15 DPA, three replicates for each samples were performed; The neighbour joining tree based on SNPs was constructed with 1,000 bootstrap replicates
Randomization	no applicable
Blinding	Blinding was not relevant for this study.

Reporting for specific materials, systems and methods

We require information from authors about some types of materials, experimental systems and methods used in many studies. Here, indicate whether each material, system or method listed is relevant to your study. If you are not sure if a list item applies to your research, read the appropriate section before selecting a response.

Materials & experimental systems

n/a	Involved in the study	
☒	☐	Antibodies
☒	☐	Eukaryotic cell lines
☒	☐	Palaeontology
☒	☐	Animals and other organisms
☒	☐	Human research participants
☒	☐	Clinical data

Methods

n/a	Involved in the study	
☒	☐	ChIP-seq
☒	☐	Flow cytometry
☒	☐	MRI-based neuroimaging
