# Supplementary figures and images for: Genome sequence of Gossypium herbaceum and genome updates of Gossypium arboreum and Gossypium hirsutum provide insights into cotton A-genome evolution
Source: Nat Genet. 2020 Apr 13;52(5):516–24. doi: 10.1038/s41588-020-0607-4 (PMC7203013; doi:10.1038/s41588-020-0607-4)

**a**

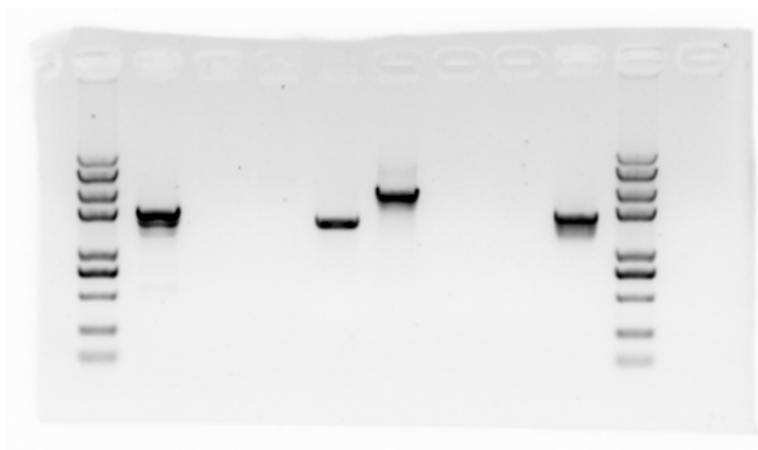

**b**

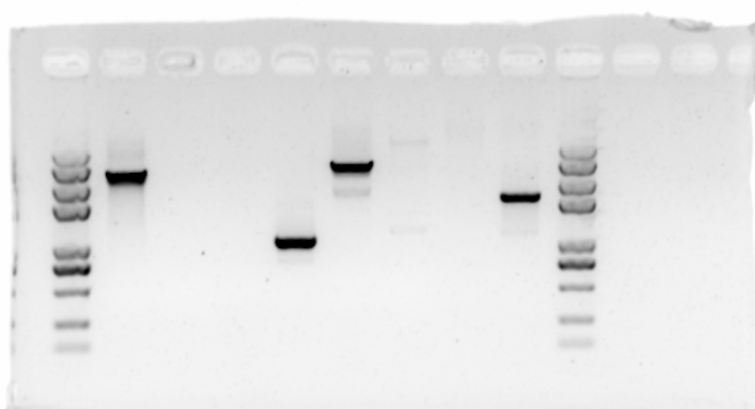

**Unprocessed gels for Extended Data Fig. 5c (a) and 5d (b).**

Supplement: Source Data Extended Data Fig. 5 — Unprocessed gels for Extended Data Fig. 5 [file 41588_2020_607_MOESM6_ESM.pdf]
